# LATS2 Positively Regulates Polycomb Repressive Complex 2

**DOI:** 10.1371/journal.pone.0158562

**Published:** 2016-07-19

**Authors:** Kosuke Torigata, Okuzaki Daisuke, Satomi Mukai, Akira Hatanaka, Fumiharu Ohka, Daisuke Motooka, Shota Nakamura, Yasuyuki Ohkawa, Norikazu Yabuta, Yutaka Kondo, Hiroshi Nojima

**Affiliations:** 1 Department of Molecular Genetics, Research Institute for Microbial Diseases, Osaka University, Suita City, Osaka, Japan; 2 Department of Epigenomics, Nagoya City University Graduate School of Medical Sciences, Nagoya City, Aichi, Japan; 3 DNA-chip Development Center for Infectious Diseases, Research Institute for Microbial Diseases, Osaka University, Suita City, Osaka, Japan; 4 Department of Infection Metagenomics, Genome Information Research Center, Research Institute for Microbial Diseases, Osaka University, Suita City, Osaka, Japan; 5 Department of Advanced Medical Initiatives, Graduate School of Medical Sciences, Kyushu University, Fukuoka City, Fukuoka, Japan; Michigan State University, UNITED STATES

## Abstract

LATS2, a pivotal Ser/Thr kinase of the Hippo pathway, plays important roles in many biological processes. LATS2 also function in Hippo-independent pathway, including mitosis, DNA damage response and epithelial to mesenchymal transition. However, the physiological relevance and molecular basis of these LATS2 functions remain obscure. To understand novel functions of LATS2, we constructed a *LATS2* knockout HeLa-S3 cell line using TAL-effector nuclease (TALEN). Integrated omics profiling of this cell line revealed that *LATS2* knockout caused genome-wide downregulation of Polycomb repressive complex 2 (PRC2) and H3K27me3. Cell-cycle analysis revealed that downregulation of PRC2 was not due to cell cycle aberrations caused by *LATS2* knockout. Not LATS1, a homolog of LATS2, but LATS2 bound PRC2 on chromatin and phosphorylated it. LATS2 positively regulates histone methyltransferase activity of PRC2 and their expression at both the mRNA and protein levels. Our findings reveal a novel signal upstream of PRC2, and provide insight into the crucial role of LATS2 in coordinating the epigenome through regulation of PRC2.

## Introduction

Large tumor suppressor 2 (LATS2), a pivotal Ser/Thr kinase of the Hippo signaling pathway, plays important roles in many biological processes, including normal development and tumorigenesis [1]. In canonical Hippo signaling, LATS2 and its homolog LATS1 phosphorylate YAP1 and WWTR1 (also known as YAP and TAZ, respectively), transcription coactivators involved in cell proliferation. Phosphorylation inhibits the function of these proteins by promoting their cytoplasmic retention and degradation, thereby governing contact inhibition, and dysregulation of this process is related to tumor progression. LATS2 also functions as a hub for many other tumor-suppressive signaling pathways, such as the tetraploidy checkpoint [2], G1/S checkpoint [3], and DNA-damage response [4–6]. LATS2 shows distinct subcellular localization depending on its phosphorylation state during the cell cycle; it also localizes to the nucleus [7, 8]. The nuclear LATS2 performs both kinase-dependent and -independent functions in collaboration with a wide range of transcriptional regulators, including TP53, SNAI1, AR, and CTNNB1/BCL9 [9–12], and thereby contributes to regulation of pluripotency and maintenance of the dedifferentiated state [13, 14]. However, the physiological relevance of these LATS2 functions to non-canonical Hippo signaling remains poorly understood.

Polycomb repressive complex 2 (PRC2) catalyzes di- and tri-methylation of histone H3 at lysine 27 (H3K27me2/3) and forms Polycomb domains involved in gene silencing [15–18]. PRC2 is composed of three core components, EZH2, EED, and SUZ12, along with accessory factors including RbAp46/48 and AEBP2. PRC2-mediated gene silencing plays an important role in maintenance of stemness and normal development [19, 20], and PRC2 is dysregulated in several types of cancers [21]. Thus, PRC2 and its epigenetic signatures represent promising therapeutic targets for tumors with specific mutations or alterations [22, 23]. In order to develop more precise tumor treatments, it is essential to elucidate the pertinent upstream signals and their spatiotemporal regulation at the molecular level. Indeed, recent studies uncovered several aspects of the post-translational regulation of PRC2 components and the molecules with which they collaborate, including non-coding RNAs.

In this study, we generated *LATS2* knockout (KO) HeLa-S3 cells to elucidate a novel LATS2 function using TALEN-mediated genome editing. Genome-wide profiles using transcriptome and epigenome analysis of *LATS2* KO cells revealed that *LATS2* KO caused a deleterious effect on global H3K27me3 integrity. Here, we show a novel functional link between LATS2 and PRC2.

## Results

### TALEN-mediated knockout of *LATS2* gene in HeLa-S3 cells

**Fig 1:**
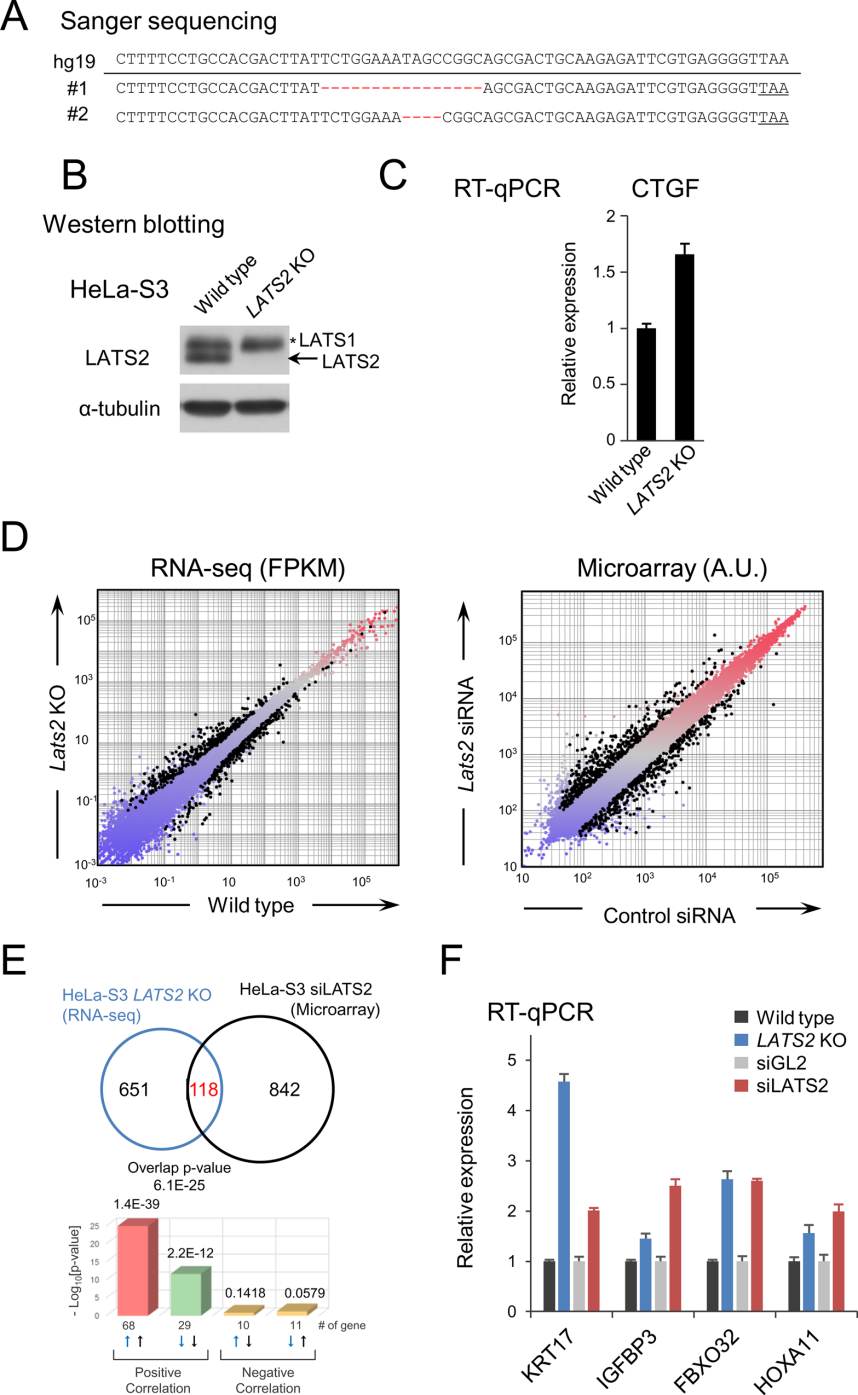
Construction of *LATS2* KO HeLa-S3 cells. (**A**) Genomic sequences of the wild type *LATS2* locus (hg19) and the *LATS2* KO mutations generated in HeLa-S3 cells. The TALEN-targeted regions of the genome were amplified by genomic PCR, the PCR products were sub-cloned, and each clone was subjected to Sanger sequencing. (**B**) Confirmation of *LATS2* KO by western blotting. The anti-LATS2 polyclonal antibody used recognizes the N-termini of both LATS2 and LATS1. Arrow represents LATS2 signals. ‘*LATS1’ indicates LATS1 signals. (**C**) Gene expression analysis of *CTGF*, which is under the control of YAP/TAZ, showing perturbation of the intrinsic Hippo signal. RT-qPCR was performed in two independent experiments, and mRNA levels were normalized to *ACTB*; Error bars show standard deviation (SD). (**D**) Left: Scatter plot of RNA-seq data comparing *LATS2* KO and wild type HeLa-S3 cells. DEGs (≥2-fold, p-value <0.05) are highlighted in black dots. Right: Scatter plot of microarray data comparing LATS2 knockdown and control siRNA HeLa-S3 cells. DEGs (≥1.4-fold, probes expressing in both samples [i.e., ‘wellAboveBG-FLAG’ is TRUE] are highlighted in black dots. (**E**) Significant overlap of DEGs in *LATS2* KO HeLa-S3 cells and HeLa-S3 cells treated with siRNA targeting LATS2. DEGs in *LATS2* KO and siLATS2 HeLa-S3 cells were subjected to NextBio analysis. Venn diagrams show the number of common and unique genes in both sets. Bar plots show the significance of overlap in each direction. (**F**) Gene expression analysis for a series of DEGs in *LATS2* KO HeLa-S3 cells and LATS2 knockdown HeLa-S3 cells. RT-qPCR was performed in two independent experiments, and the levels of each transcript were normalized to *ACTB*; Error bars show SD.

To explore the cellular functions and/or signals that potentially fluctuate in LATS2 dependent fashion, we established *LATS2* knockout (KO) HeLa-S3 strains by inducing TALEN-mediated double-strand breaks, followed by successive generation of frameshift mutations by non-homologous end joining [24]. Transient expression of TALENs targeting the *LATS2* gene locus (Forward: hg19_chr13:21,620,130–21,620,148; Reverse: hg19_chr13:21,620,095–21,620,113) resulted in successful knockout of *LATS2* (genomic: Fig **1A**, protein level: Fig **1B**). Expression analysis of *CTGF* (1.6-fold increase upon *LATS2* KO), a downstream target gene of the Hippo pathway that should negatively correlate with LATS2 kinase activity, confirmed downregulation of intrinsic LATS2 expression (Fig **1C**). To confirm the dependency of the overall expression profile on LATS2 and exclude the possibility of obvious off-target effects of the TALEN system, we calculated the correlation between differentially expressed genes (DEGs) in *LATS2* KO HeLa-S3 cells and siRNA-mediated LATS2-knockdown cells. Although we used different analytical platforms (RNA-sequencing (RNA-seq) for *LATS2* KO cells, microarray for the knockdown study) (summarized in Fig **1D**), a significant portion of DEGs (15%; 118 of 769 genes) overlapped and positively correlated (p = 6.1E-25, Fisher’s exact test) between the two types of cells (Fig **1E**; DEGs are listed in **S1** and **S2** Tables). Some DEGs detected in both cell types were also validated by RT-qPCR analysis (Fig **1F**). Following these validation, we subjected this *LATS2* KO HeLa-S3 cell line to further analysis.

### *LATS2* KO causes downregulation of H3K27me3

**Fig 2:**
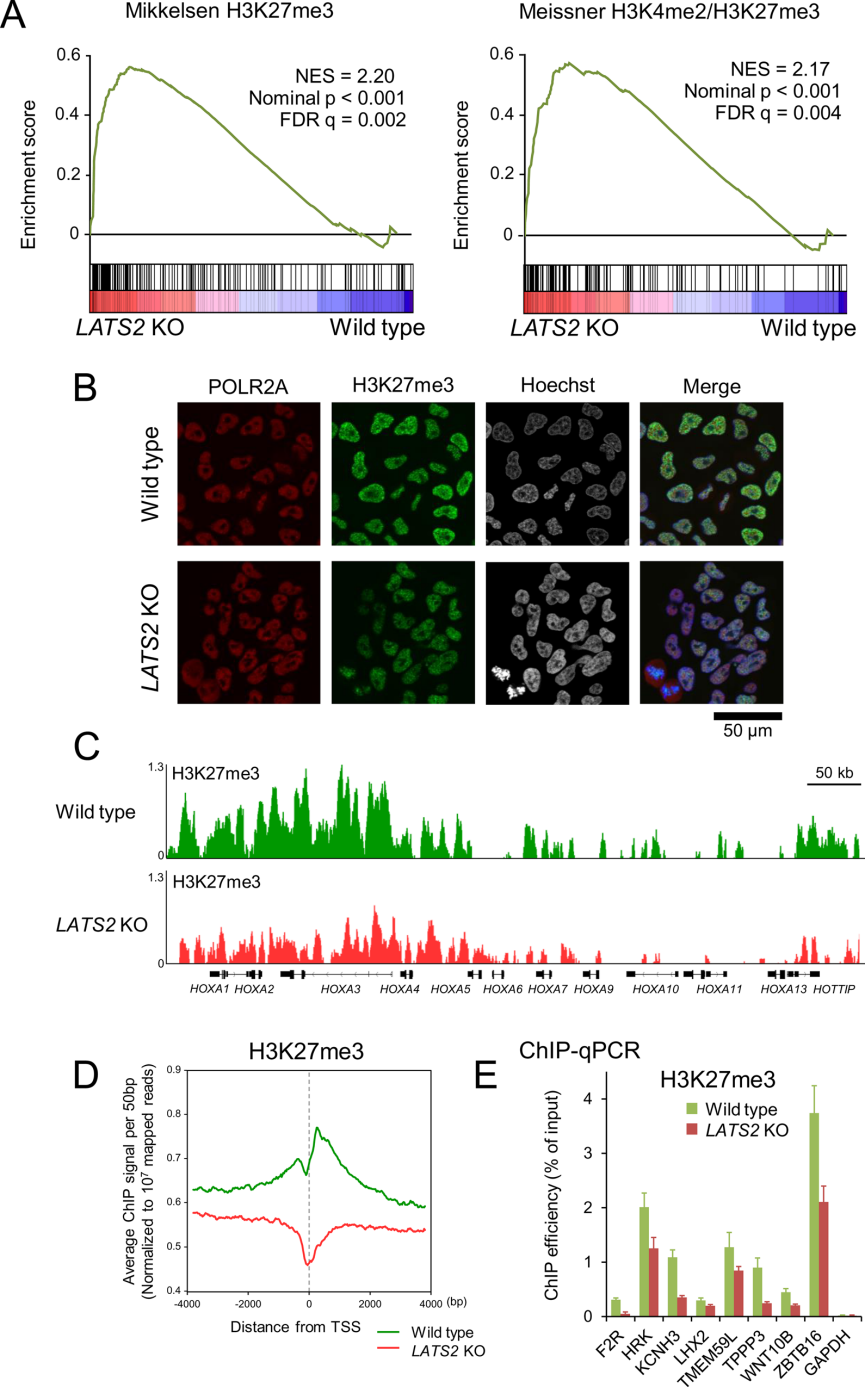
Dysregulation of H3K27me3 regulation upon *LATS2* KO. (**A**) GSEA of *LATS2* KO HeLa-S3 cells for gene sets related to H3K27me targets. Genes are ranked by fold change in a RNA-seq experiment (KO vs. wild type). A positive enrichment score indicates higher expression after *LATS2* KO. (**B**) Immunofluorescence showing reduction of global H3K27me3 in *LATS2* KO HeLa-S3 cells relative to the wild type. RNAPII and nuclei were counterstained. Bar indicates 50 μm. (**C**) Snapshots of ChIP-seq traces for H3K27me3 in the wild type HeLa-S3 (green) and *LATS2* KO cells (red). The *HOXA* gene cluster is depicted as a representative locus showing a reduction of H3K27me3 following *LATS2* KO. (**D**) Aggregate plots of H3K27me3 ChIP-seq signals centered at TSSs of all RefSeq genes in wild type HeLa-S3 (green) and *LATS2*-KO cells (red). (**E**) ChIP-qPCR analysis for H3K27me3 on a series of known PRC2 target loci. All ChIP experiments were performed at least twice independently; error bars show SD.

Next, we sought to identify the gene signatures associated with *LATS2* KO. Using RNA-seq data, we performed gene set enrichment analysis (GSEA) [25] to extract cellular functions associated with LATS2 from ‘C2 cgp gene sets collection’. This collection includes gene sets representing expression signatures of genetic and chemical perturbations in many previous omics-based studies. *LATS2* KO cells were positively correlated with high expression of epigenetically silenced genes, especially H3K27me3-marked genes (p-value < 0.001) (Fig **2A**; top 25 gene sets are listed in **S3** Table). To confirm the impact of *LATS2* KO on the level of H3K27me, we performed immunofluorescence imaging. Consistent with the positive correlation of LATS2 with H3K27me3 in GSEA, *LATS2* KO decreased the H3K27me3 level (Fig **2B**). To more precisely determine the impact of LATS2 on epigenetic landscapes, we next performed high-throughput sequencing of ChIP-enriched DNA (ChIP-seq) for H3K27me3 marks. Consistent with the results shown in Fig 2A and 2B, H3K27me3 levels were reduced at target loci (*HOXA* locus as a representative; Fig **2C**), as well as at promoter regions, on a genome-wide scale (aggregated for all transcription start sites [TSSs]; Fig **2D**). In order to validate the results above, we examined H3K27me3 levels at known PRC2 target loci, i.e., genes that have the H3K27me3 mark and are bound by SUZ12 and EED on their promoters in human embryonic stem cells [26]. Although the magnitude of fluctuations determined by ChIP-qPCR varied, we observed an overall trend toward downregulation of H3K27me3 levels at these loci (Fig **2E**). These results suggest that LATS2 exerts a positive effect on PRC2 function, and that deletion of LATS2 therefore causes genome-wide downregulation of H3K27me3.

### *LATS2* affects H3K27me3 genome-wide in a kinase-dependent fashion

**Fig 3:**
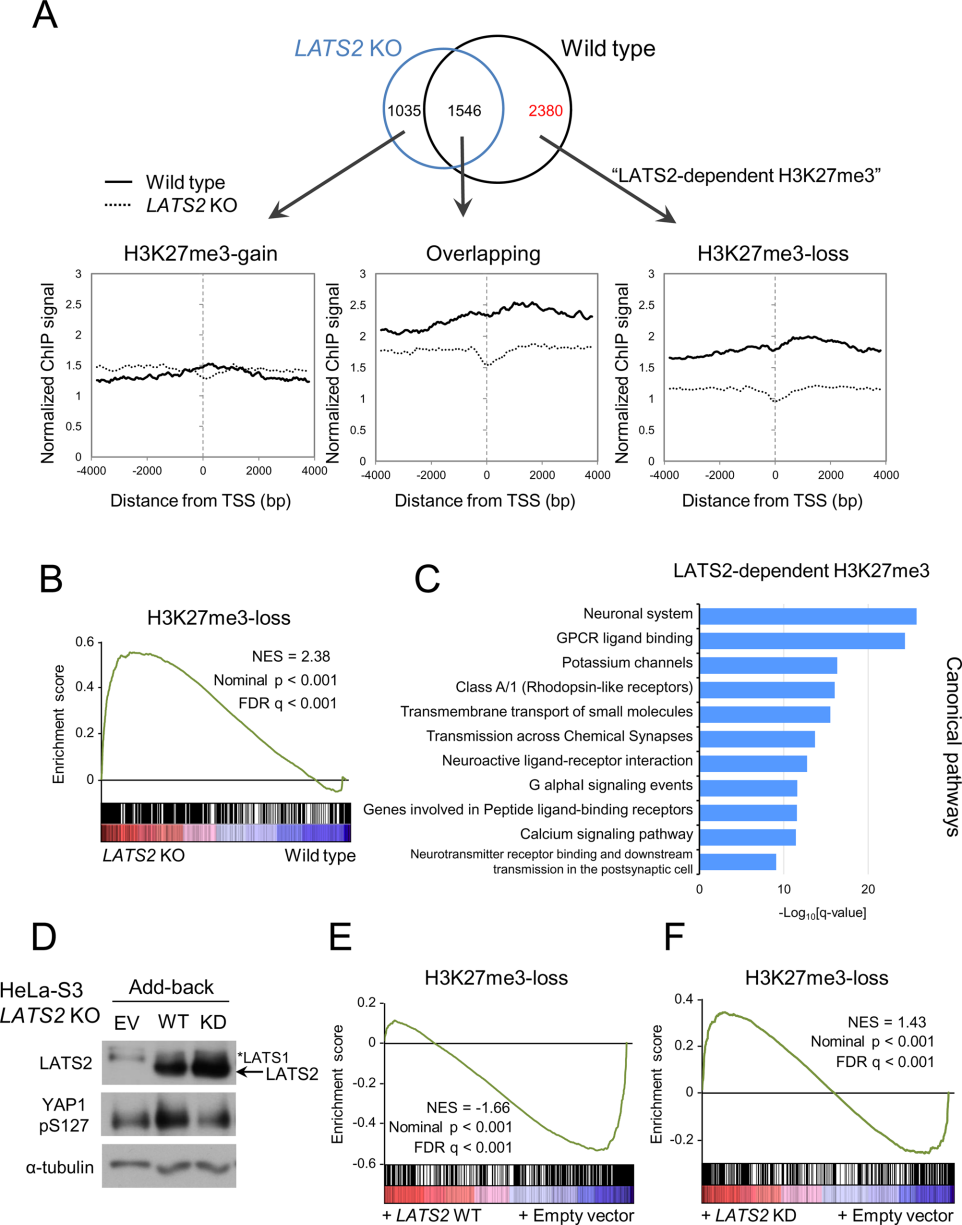
ChIP-seq profiling for LATS2-dependent H3K27me3 targets. (**A**) Top: Venn diagram showing the overlap of genes with H3K27me3 peaks within ±5 kb of the TSS in wild type (blue) and *LATS2* KO HeLa-S3 cells (black). Bottom: Aggregate plots of H3K27me3 ChIP-seq signals centered at TSSs of RefSeq genes for each module of wild type (solid line) and *LATS2* KO HeLa-S3 cells (dashed line). (**B**) GSEA of *LATS2* KO HeLa-S3 cells, for genes with *LATS2* KO–responsive H3K27me3 marks in their promoters. Genes are ranked by fold change, derived from the RNA-seq experiment (KO vs. wild type). A positive enrichment score indicates increased expression after *LATS2* KO. (**C**) GO enrichment analysis of canonical pathways for LATS2-dependent H3K27me3 targets. The x-axis represents statistical significance. The y-axis represents gene sets belonging to 'canonical pathways' from MSigDB (Broad Institute). (**D**) Western blotting of whole cell lysate of rescued *LATS2* KO HeLa-S3 cells. Phosphorylated YAP was blotted as an indicator of LATS2 kinase activity. EV, empty vector. WT, kinase active. KD, kinase-inactive mutant. (**E**) GSEA of rescued *LATS2* KO HeLa-S3 cells, for genes with *LATS2*-KO–responsive H3K27me3 marks in their promoters. Left: cells rescued by kinase active (WT) LATS2. Right: cells rescued by kinase-inactive (KD) LATS2. Genes are ranked by fold change, derived from the RNA-seq experiment (LATS2 add-back vs. empty vector). A positive enrichment score indicates increased expression upon LATS2 add-back.

The aggregate plots in Fig 2D suggested an existence of unidentified genes fluctuated strongly upon *LATS2* KO. To investigate the chromatin state in more detail, we divided the genes into three groups depending on their H3K27me3 status: 1) H3K27me3-overlap, genes possessing peaks that were called by the MACS software [27] within +/- 5 kb of each TSS in both wild type and *LATS2* KO cells (1546 genes); 2) H3K27me3-loss, genes possessing peaks only in wild type cells (2380 genes), and 3) H3K27me3-gain, genes possessing peaks only in *LATS2* KO cells (1035 genes) (Fig **3A**, upper panel). The aggregate analysis for each module revealed that the H3K27me3-gain module maintained the same level of H3K27me3 between wild type and *LATS2* KO cells (Fig **3A**, lower panels). This observation suggests that *LATS2* KO has mostly inhibitory effects on H3K27me3 maintenance, and that sensitivity to this effect varies across the genome. To examine the LATS2 dependency of transcriptome changes accompanied by epigenetic changes, we performed GSEA on the H3K27me3-loss module, the module most sensitive to *LATS2* KO (the genes are listed in **S4** Table). Genes in this module were significantly upregulated upon *LATS2* KO (p-value < 0.001) (Fig **3B**). To examine the potential functional relevance of this module, we next calculated the enrichment of gene ontology (GO) terms using ‘Canonical pathways’ in the NextBio statistical platform [28] for genes in the H3K27me3-loss module. Indeed, the H3K27me3-loss module exhibited significant enrichment in GO terms related to neural functions (Fig **3C**). This intriguing enrichment might reflect the specific function of this module in these contexts or tissues (discussed in more detail in **S1** File). We next performed an add-back rescue experiment by constructing cell lines in which LATS2 was stably expressed (Fig **3D**). GSEA of RNA-seq data of the rescued *LATS2* KO cells revealed that cells expressing wild type LATS2 (WT), but not a kinase-dead form of LATS2 (KD), re-repressed the genes in the H3K27me3-loss module (WT: enrichment score = -0.583, KD: enrichment score = 0.344, p-value < 0.001) (Fig **3E** and **3F**). This observation supports the idea that LATS2 depletion causes downregulation of PRC2 and H3K27me3 signatures, and that this phenotype is dependent on LATS2 kinase activity.

### *LATS2* knockout causes downregulation of PRC2 at both the mRNA and protein levels

**Fig 4:**
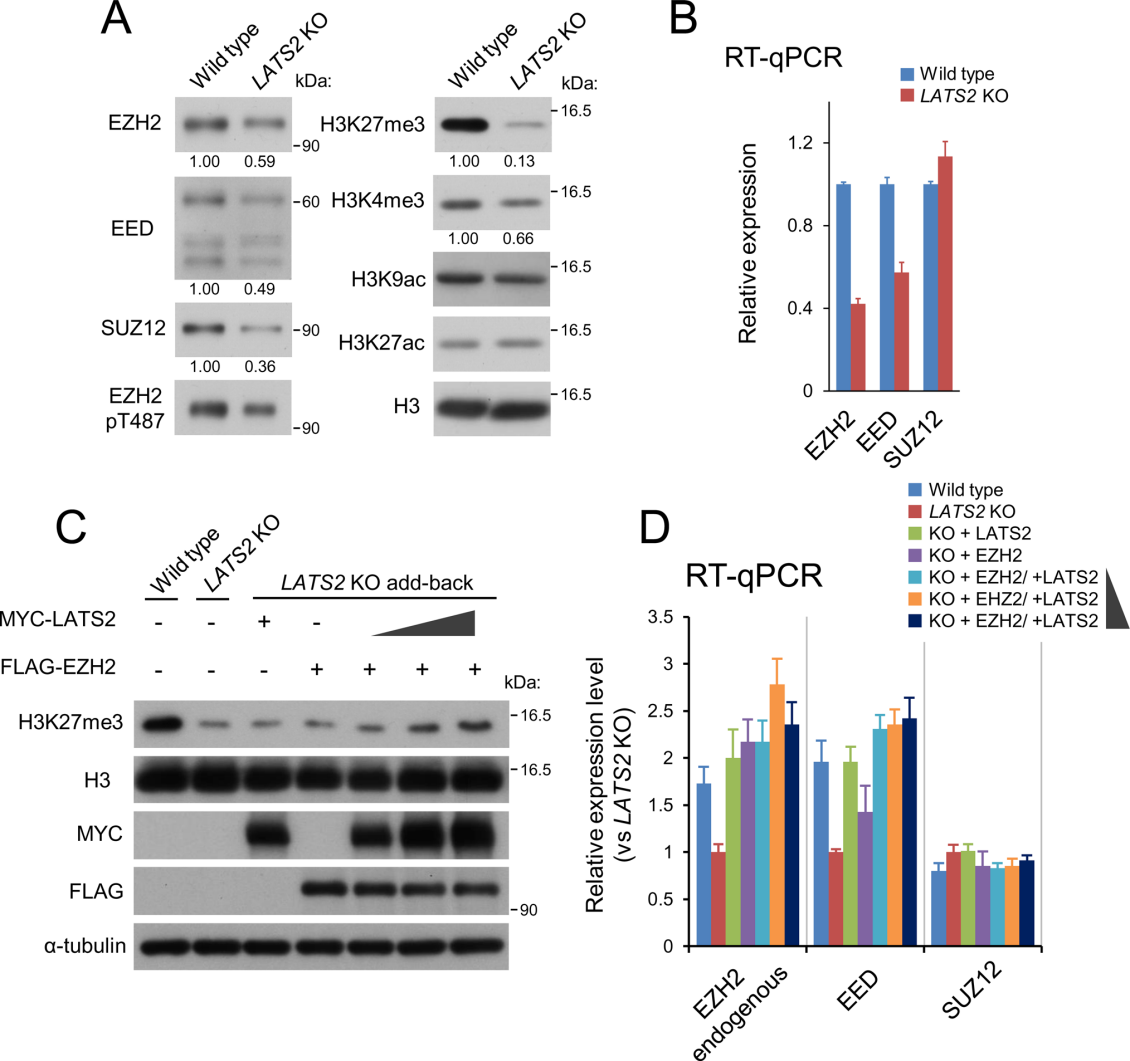
*LATS2* KO downregulates PRC2 at both the protein and mRNA levels. (**A**) Polycomb components and major histone marks following *LATS2* KO. The chromatin-bound fraction was subjected to western blotting. (**B**) Gene expression analysis for the core components of PRC2: EZH2, EED, and SUZ12. RT-qPCR was performed in two independent experiments, and transcript levels were normalized against *ACTB*; Error bars show SD. (**D**) Western blotting of rescued *LATS2* KO cells by transient overexpression of MYC-tagged LATS2 and/or FLAG-tagged EZH2. The synergetic effects and the dose dependency of LATS2 were evaluated by increasing amounts of LATS2. (**E**) Gene expression analysis for the core components of PRC2 in the same setup in (D). The expression level of endogenous EZH2 was quantified by using primers targeted 3’UTR region of mRNA. RT-qPCR was performed in two independent experiments, and transcript levels were normalized against *ACTB*; Error bars show SD.

Next, to determine how *LATS2* knockout causes genome-wide downregulation of H3K27me3, we characterized the state of PRC2 in *LATS2*-KO HeLa-S3 cells. Immunoblotting of the solubilized chromatin fraction revealed that the levels of three core components of PRC2 (EZH2, SUZ12, and EED) were reduced in *LATS2*-KO cells (Fig **4A**). Consistent with the ChIP-seq analysis, immunoblotting of whole chromatin revealed a significant reduction in H3K27me3 (13% of wild-type level) (Fig **4A**). RT-qPCR revealed that PRC2 was also downregulated at a transcriptional level; specifically, expression of EZH2 (42% of the wild-type level) and EED (57%), but not SUZ12, was reduced upon *LATS2* knockout (Fig **4B**). Moreover, to confirm the effects of LATS2 and EZH2 on genome-wide H3K27me3 level and the transcription level of PRC2, we performed another add-back rescue experiment. Transient add-back of LATS2 and/or EZH2 revealed that co-overexpression of LATS2 and EZH2 restored the reduction of H3K27me3 in *LATS2*-KO cells in a LATS2 dose–dependent fashion, although transient overexpression of LATS2 or EZH2 alone exerted no effect on global H3K27me3 level (Fig **4C**). It should also be noted that overexpression of LATS2 or EZH2 increased endogenous transcription of EZH2 and EED (Fig **4D**). These results suggest that a threshold amount of EZH2 is required to restore the global H3K27me3 level.

### Down-regulation of PRC2 in LATS2 KO cells is not due to cell cycle aberrations

**Fig 5:**
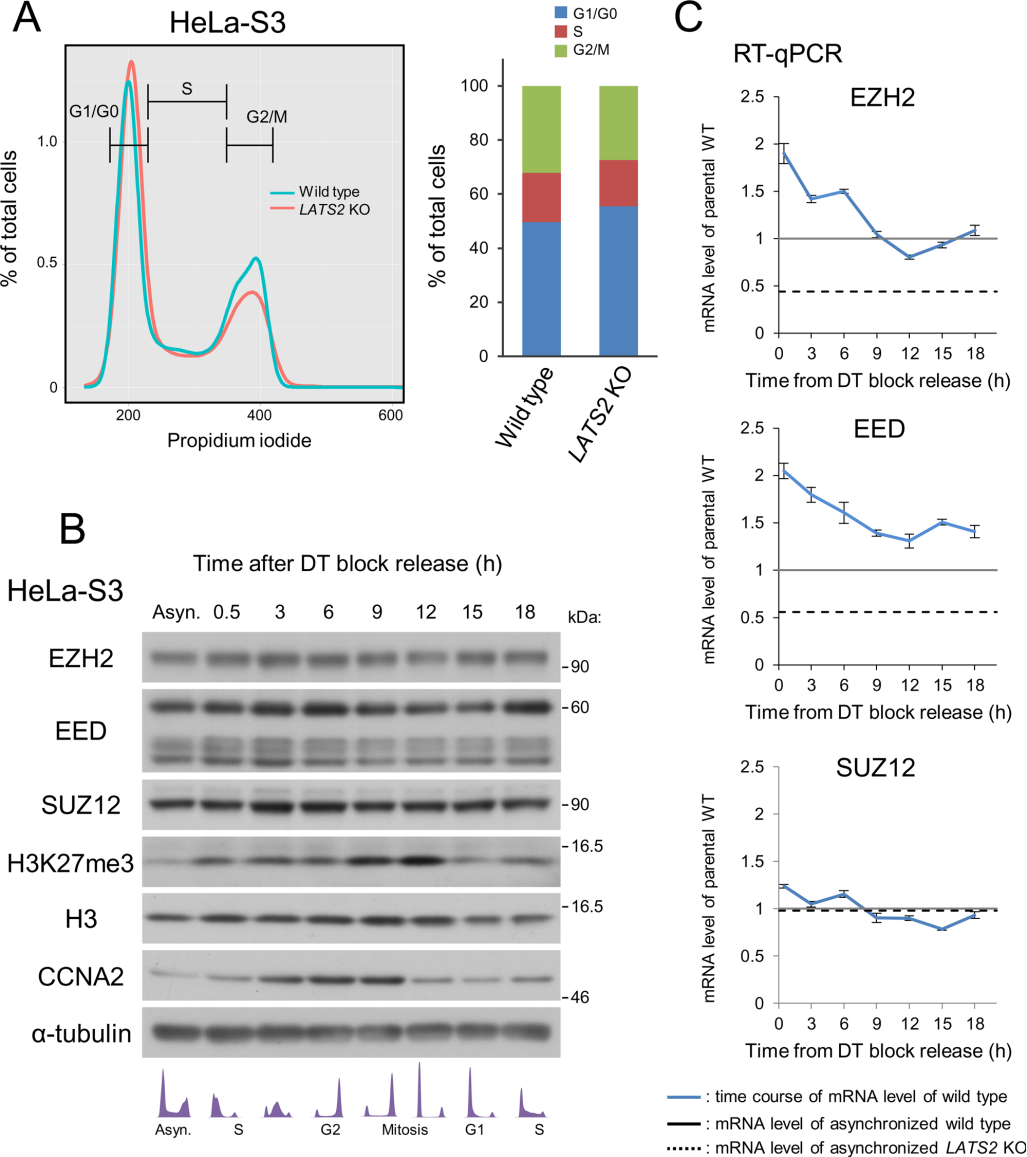
Downregulation of PRC2 upon *LATS2* KO is not due to cell cycle aberrations. (**A**) No differences in the cell cycle were observed in HeLa-S3 cells upon KO. Cell-cycle analysis by FACS showing that growing, asynchronous *LATS2* KO HeLa-S3 cells do not exhibit retention at any stage of the cell cycle. (**B**) Western blotting of PRC2 components and the H3K27me3 mark in wild type HeLa-S3 cells throughout the cell cycle. α-tubulin and H3 were used as loading controls, and CCNA2 was used as a cell-cycle indicator. A portion of the cells was analyzed by FACS, and is depicted in the bottom panel. (**C**) RT-qPCR analysis of PRC2 components in wild type HeLa-S3 cells. Black line and dotted line indicate the expression level of each gene in asynchronous wild type and *LATS2* KO HeLa-S3 cells, respectively. The expression levels of EZH2 and EED oscillate during the cell cycle but do not reach the level attained in *LATS2* KO cells. Each transcript level was normalized against *ACTB*; Error bars show SD.

Although LATS2 is a mitotic kinase involved in the G1/S and tetraploidy checkpoints [2, 3], downregulation of PRC2 was not a result of cell-cycle retention due to *LATS2* knockout, as flow cytometry analysis revealed no significant difference in cell-cycle progression between asynchronous wild type and *LATS2*-KO cells (Fig **5A**).

Furthermore, in wild type HeLa-S3 cells, PRC2 expression was not reduced at either the protein or mRNA level during time-course monitoring, though the level of H3K27me3 oscillates during the cell cycle, possibly reflecting an increase in the chromatin content per cell followed by DNA replication. (Fig **5B** and **5C**). These observations suggest that down-regulation of PRC2 and H3K27me3 upon LATS2 knockout is not due to cell-cycle aberrations caused by LATS2 depletion. One possible explanation of the molecular mechanisms underlying transcriptional regulation of EZH2 and EED is that other epigenetic mechanisms are perturbed by PRC2 dysfunction upon *LATS2* KO. Indeed, we detected moderate genome-wide reduction of H3K4me3 (Fig A in **S1** File). Consistent with the RT-qPCR results in Fig 4B, further analysis revealed a trend toward downregulation of H3K4me3 at *EZH2* and *EED*, but not the *SUZ12* locus, in *LATS2* KO cells (Fig A in **S1** File).

### LATS2 kinase affects histone methyltransferase activity of PRC2

**Fig 6:**
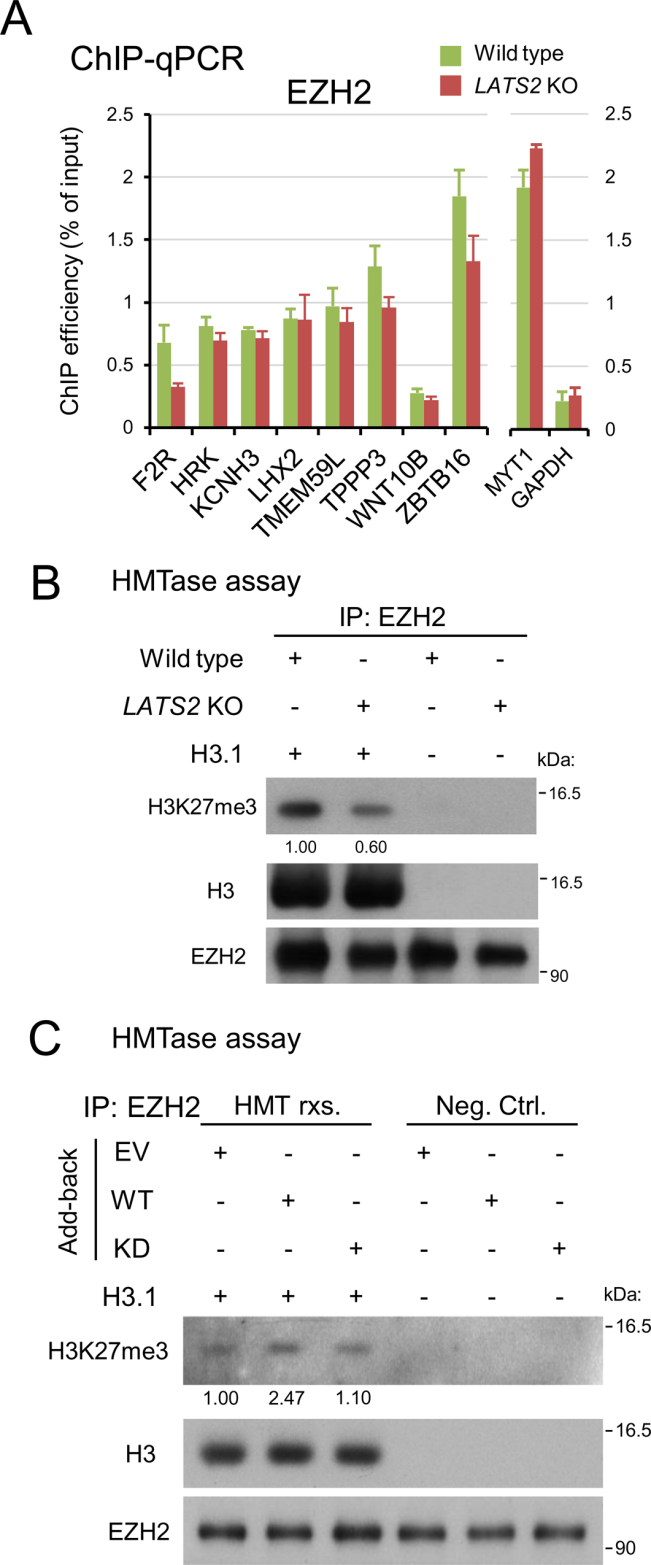
LATS2 affects HMTase activity of PRC2 in kinase dependent fashion. (**A**) ChIP-qPCR analysis for EZH2 in a series of known PRC2 targets assessed in Fig 2E. The MYT1 locus and GAPDH locus are positive and negative control regions for EZH2, respectively. All ChIP experiments were performed at least twice independently; error bars show SD. (**B**) *In vitro* HMTase assay with immunoprecipiated EZH2 of *LATS2* KO cells. Endogenous PRC2 was purified by immunoprecipitation of active chromatin fraction of each cell line. The background H3K27me3-level was validated in no-substrate setup. The amount of EZH2 protein in IP-input was titrated beforehand, the equivalent EZH2 between samples was used for HMTase reaction. Each H3K27me3 level was normalized by the signal of immunoprecipitated EZH2. (**C**) *In vitro* HMTase assay with immunoprecipiated EZH2 of each add-back cell line. Endogenous PRC2 was purified by immunoprecipitation of active chromatin fraction of each add-back cell line. The background H3K27me3-level was validated in no-substrate setup (Negative control; Neg. Ctrl.), and HMTase activity of each add-back cell line was evaluated by western blotting (HMTase reaction; HMT rxs.). EV, empty vector. WT, kinase active. KD, kinase-inactive mutant. Each H3K27me3 level was normalized by the signal of immunoprecipitated EZH2.

Inhibition of histone methyltransferase (HMTase) activity of EZH2 by small molecules such as 3-deazaneplanocin A not only reduces the catalytic ability of this protein, but also downregulates its transcription via negative-feedback mechanisms [29], suggesting downregulation of HMTase activity upon *LATS2* KO. Indeed, the significant reduction in H3K27me3 level could not be explained by reduction of PRC2 expression alone. ChIP-qPCR analysis of EZH2 revealed that the magnitude of reduction in the occupancy of EZH2 at PRC2 target loci (analyzed in Fig 2E) was relatively moderate (Fig **6A**). To determine whether HMTase activity was also affected in *LATS2* KO cells, we performed *in vitro* HMTase assays using recombinant H3.1 and endogenous immunoprecipitated EZH2 from each sample. The results revealed a decrease in methyltransferase activity in *LATS2* KO cells (60% of wild-type activity; normalized to the amount of EZH2 protein in each tube) (Fig **6B**). Further *in vitro* HMTase assays using the add-back cell lines revealed that HMT activity was affected by LATS2 kinase activity (2.3-fold higher in the WT than in the KD mutant) (Fig **6C**). These results suggest that HMTase activity of EZH2 is positively regulated by LATS2 kinase.

### LATS2 associates with PRC2 and phosphorylates it on chromatin

**Fig 7:**
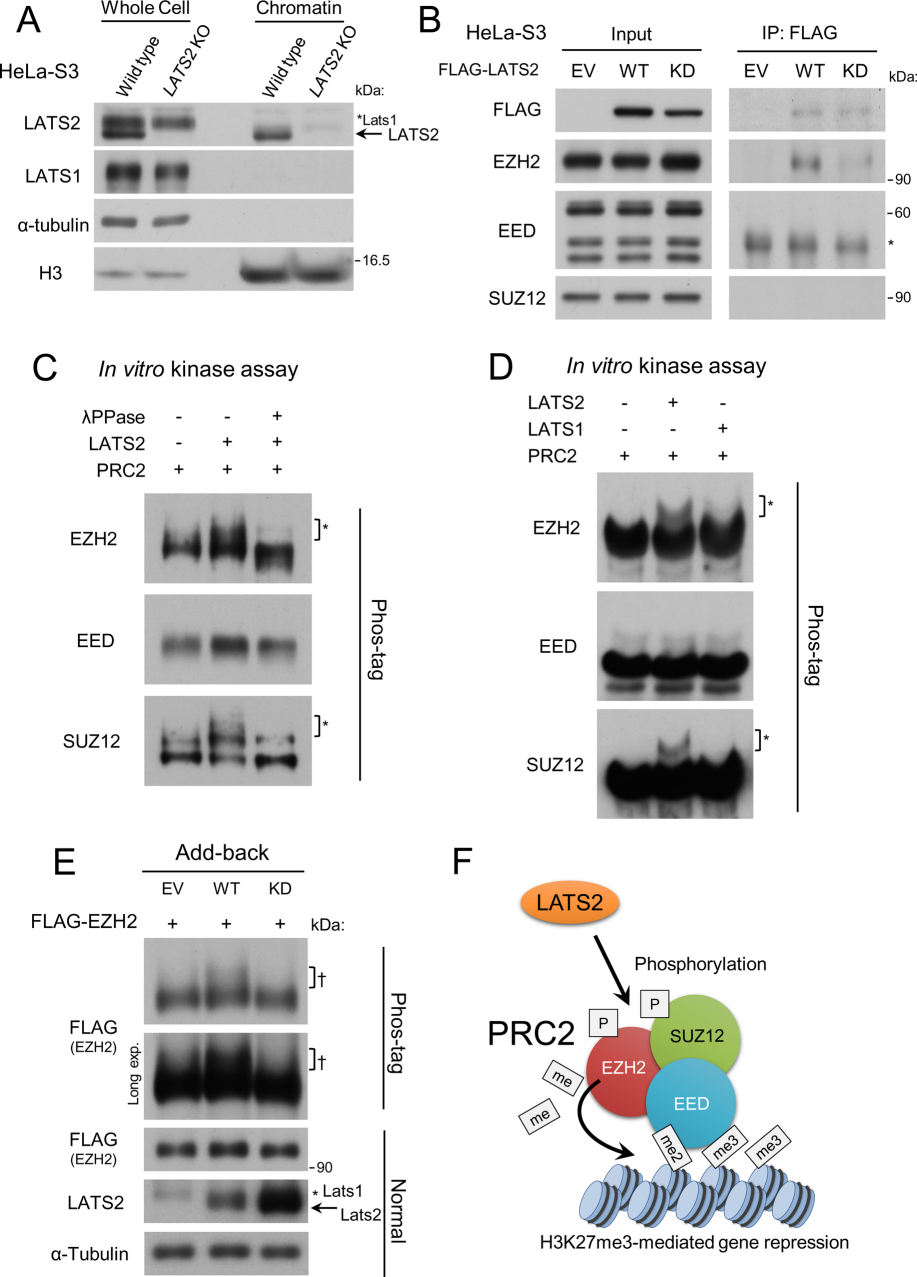
LATS2 associates with PRC2 and phosphorylates EZH2 on chromatin. (**A**) Western blotting of LATS2 in *LATS2* KO HeLa-S3 cells. Arrow represents LATS2 signals. ‘*LATS1’ indicates LATS1 signals. (**B**) Co-immunoprecipitation of endogenous PRC2 with FLAG-tagged LATS2 from transiently transfected HeLa-S3 cells. Input represents 10% of the total solubilized chromatin fraction used for each immunoprecipitation. Right, anti-FLAG precipitates immunoblotted to detect FLAG-tagged proteins and endogenous PRC2. EV, empty vector. WT, kinase active. KD, kinase-dead mutant. The asterisk represents non-specific signals. (**C**) Phos-tag–based *in vitro* kinase assay demonstrating that LATS2 can phosphorylate PRC2. Recombinant PRC2 complex was incubated with recombinant LATS2 in the presence of ATP. Phosphorylation of PRC2 components was assessed by western blotting in the presence of Phos-tag. The asterisks indicate phosphorylation-dependent motility shifts. Phosphatase treatment demonstrated that the motility shift was dependent upon phosphorylation. (**D**) Phos-tag–based *in vitro* kinase assay including recombinant LATS1 kinase. (**E**) Phos-tag–based western blotting of add-back cells overexpressing FLAG-tagged EZH2. The dagger (†) indicates the phosphorylation-dependent motility shift. The arrow indicates LATS2 signals. ‘*LATS1’ indicates LATS1 signals. EV, empty vector. WT, kinase active. KD, kinase-dead mutant. (**F**) Model of the LATS2 signal that supports PRC2 functions.

The data presented above suggest that LATS2 somehow affects PRC2 function. One simple explanatory model is that LATS2 phosphorylates PRC2 on chromatin, thereby supporting or specializing its function. Indeed, previous studies suggested that EZH2 undergoes several post-translational modifications including phosphorylation. To investigate this possibility, we first validated whether LATS2 localizes on chromatin. Immunoblotting analysis of the chromatin-bound fraction revealed that LATS2, but not LATS1, was present in the chromatin fraction of HeLa-S3 and MDA-MB231 cells (Fig **7A**, Fig B in **S1** File), consistent with a previous study showing that LATS2, but not LATS1, binds to chromatin along with effectors of Wnt signaling [12]. This result also suggests that LATS2 exerts some functions on chromatin that are distinct from those of LATS1. To determine whether LATS2 associates with PRC2, we immunoprecipitated LATS2 and PRC2 core components. Because the absolute level of endogenous LATS2 on chromatin is very low relative to that of PRC2, we performed the immunoprecipitation experiment in HeLa-S3 cells overexpressing FLAG-tagged LATS2. We detected an association between FLAG-tagged LATS2 and endogenous EZH2 (Fig **7B**).

To determine whether LATS2 can phosphorylate PRC2, we performed *in vitro* kinase assays using a Phos-tag–based technique. EZH2 and SUZ12 exhibited a phosphorylation-dependent motility shift in the presence of LATS2, but not LATS1 (Fig **7C** and **7D**). Based on the immunoprecipitation results, we performed further analyses of EZH2. To determine whether phosphorylation of EZH2 is affected in a kinase-dependent fashion in living cells, we performed an *in vivo* kinase assay using add-back cells overexpressing FLAG-tagged EZH2. We detected a motility shift in add-back cells expressing WT LATS2 but not KD LATS2 (Fig **7E**). In the cellular Phos-tag analysis, the shifted band was broad and smeared (Fig **7E**), suggesting that LATS2 may phosphorylate EZH2 on multiple sites *in vivo*. Based on these findings, we conclude that LATS2 associates with PRC2 on chromatin and phosphorylates it to regulate its functions (Fig **7F**).

### An insight of biological functions of LATS2 –PRC2 axis in normal development and tumorigenesis

Finally, we attempted to examine whether the LATS2–responsible PRC2 signal in HeLa-S3, i.e., H3K27me3-loss module, was fluctuated in specific cells and/or tissues. Although genes in this module were derived from analysis of HeLa-S3, a cervical cancer cell line, we found a series of functional correlation of this module with neural differentiation processes (Figs C–E in **S1** File). Indeed, further analysis of glioblastoma multiforme (GBM), the most common and most aggressive malignant primary brain tumor, supports the possibility of LATS2 for tumorigenesis through PRC2 (Figs F and G in **S1** File). Consistent with these observations, we previously reported that *Lats2*-null mice exhibit developmental defects in the central nervous system [30]. Furthermore, our microarray analysis of *Lats2* KO mouse embryonic fibroblasts also showed upregulation of genes under control of PRC2 (Figs H–J in **S1** File).

## Discussion

Many studies have shown that PRC2 and other epigenetic coordinators play fundamental roles in stemness maintenance, development, and tumorigenesis [19, 20]. These discoveries were made possible in large part by advances in high-throughput sequencing technologies. Accordingly, massive epigenome datasets from many cell types were generated by global projects such as the ENCODE consortium [31]. Despite the increased availability of public datasets, these data primarily consist of ‘snapshot’ images of specific targets and cell lines. On the other hand, due to the challenges of characterizing the multiple key components of each complex and searching for novel accessory components [32–37], the upstream signals and downstream specificity of these factors remain poorly understood at a molecular level. Based on many studies of epigenomic profiles, including analyses of DNA methylation patterns, histone modification states, and higher-order chromatin conformation, it is clear that many human diseases, including cancers, are associated with changes in the epigenetic landscape. Indeed, novel drugs have been developed to inhibit the enzymes that regulate the epigenetic machinery [38–42]. However, the epigenetic signatures targeted by these drugs differ across cell types and tissues. To overcome this problem, current drugs target known somatic mutations of epigenetic regulators in order to ensure specificity. In light of this situation, it is clear that understanding the upstream signals of epigenetic regulators will be necessary to achieve more effective and accurate clinical applications.

In this study, we showed that LATS2, a pivotal Ser/Thr kinase of the Hippo signaling pathway, is a novel upstream regulator of PRC2 (illustrated in Fig **7F**). The association of *Lats* kinase with Polycomb genes was first identified in *Drosophila*: a mutant of *Wts*, the Drosophila homolog of Lats1/2, phenocopies the effect of Polycomb group (PcG) mutants on dendrite neuron maintenance [43], but no further precise characterization was performed. Thus, our findings in this study expand the role of LATS2 in epigenetic coordination from *Drosophila* to higher organisms. Indeed the preliminary analyses of LATS2-dependent H3K27me3 module which was obtained by ChIP-seq of *LATS2* KO HeLa-S3 cells and cancer genomics data from TCGA (The Cancer Genome Atlas) cohorts, suggest the possibility of LATS2 –PRC2 axis in mammalian nervous system including tumorigenesis (Discussed in detail in **S1** File). An essential function of LATS2 in this tissue is also indicated by observations in knockout mice: *Lats2*-null mice exhibit embryonic lethality due to developmental defects in the central nervous system [30]. The molecular basis of this intriguing insight should be addressed in detail in the future. Downregulation of PRC2 components was observed at both the protein and mRNA levels in *LATS2* KO HeLa-S3 cells. Although the HMTase activity of PRC2 was affected by LATS2 kinase (Fig **6B** and **6C**), the molecular mechanisms underlying transcriptional regulation of EZH2 and EED remain obscure (**Fig 4B** and **4D**). ChIP-seq analysis for H3K4me3-mark suggests that active histone modification was also reduced upon *LATS2* KO (Fig A in **S1** File). The next challenge is to elucidate the crosstalk of these epigenetic mechanisms (H3K27me3 and H3K4me3 etc.) dependent on LATS2 kinase.

## Conclusions

In summary, our genome-wide analysis of *LATS2* KO HeLa-S3 cells reveals a novel functional link between LATS2 and PRC2 to maintain H3K27me3 integrity. LATS2 associates with PRC2 on chromatin and phosphorylates it. LATS2 kinase affects HMTase ability of PRC2 and also downregulates their expression at both the protein and transcription level. Taken together, our results suggest a novel role of LATS2 in maintenance of appropriate epigenetic integrity.

## Materials and Methods

### Cell culture

*Lats2*-deficient MEFs were described in our previous study [30]. MEFs, HeLa-S3, MCF7, and MDA-MB231 cells were cultured in DMEM containing 10% FBS and antibiotics (streptomycin and penicillin) at 37°C in a 5% CO2/95% air atmosphere. Cells were seeded the day before drug treatment.

### Generation of TALEN-mediated LATS2-knockout HeLa-S3 cell line

The ORFs for TALEN targeting the human LATS2 locus (Forward: hg19_chr13:21,620,130–21,620,148, and Reverse: hg19_chr13:21,620,095–21,620,113) were synthesized by GeneArt (Life Technologies, Danvers, MA, USA). The Bowtie software was used to confirm that the target sites were unique in the human genome (hg19). The coding region of this entry clone was sub-cloned into an expression vector, pDEST26 (Life Technologies, Danvers, MA, USA), using the Gateway technology. The two resultant expression constructs were transfected into HeLa-S3 cells using Lipofectamine 2000 (Life Technologies, Danvers, MA, USA). Clones derived from single cells were expanded as candidate knockout cell lines. Successful knockout of LATS2 was validated by endonuclease assay, Sanger sequencing of the target locus, and immunoblotting analysis. The primer sets used are provided in the S7 Table.

### Generation of add-back rescued cell lines derived from *LATS2* KO HeLa-S3 cells

Expression constructs encoding the *LATS2* wild type (WT) or kinase-dead (KD) (K698M) mutant were transfected into *LATS2*-KO HeLa-S3 cells using Lipofectamine 2000 (Life Technologies, Carlsbad, CA, USA). After 2 weeks of selection with 800 μg/ml G418 (Nacalai Tesque, Kyoto, Japan), clones derived from single cells were expanded as candidate add-back clones. Stable expression of the exogenous *LATS2* genes was validated by western blotting analysis.

### Plasmids

3xFLAG and 6xMYC-tagged human LATS2 wild type (WT) and kinase-dead (KD, K698M) were described in our previous studies [5, 6]. pcDNA3.1-human LATS2 WT and KD plasmids for generation of stable expressing cell lines were constructed by subcloning each ORF into pcDNA3.1+AscI, a modified version of pcDNA3.1(+). cDNA of human EZH2 was PCR amplified from HEK293T cDNA pool and ligated into the *Asc*I and *Not*I sites of p3Flag+AscI, a modified version of p3xFLAG-CMV-7.1. All PCR amplified sequences were confirmed by Sanger DNA sequencing.

### Antibodies

The antibodies used for western blotting, co-immunoprecipitation and ChIP experiments in this study are provided in S9 Table in detail.

### Western blotting

For western blotting, protein samples were prepared by lysing cells in RIPA lysis buffer (50 mM Tris-HCl [pH 8.0], 150 mM sodium chloride, 0.5% (w/v) sodium deoxycholate, 0.1% [w/v] sodium dodecyl sulfate, and 1.0% [w/v] Nonidet P-40, plus protease and phosphatase inhibitors). Equal amounts of proteins from cell lysates were denatured in sample buffer, subjected to SDS-PAGE, and transferred to PVDF membranes (GE Healthcare, Little Chalfont, UK). The membranes were blocked in 5% nonfat milk or BSA in TBS-T at room temperature for 1 h with gentle shaking. The membranes were then immunoblotted with specific primary antibodies and horseradish peroxidase–conjugated secondary antibodies (Cell Signaling Technology, Danvers, MA, USA), and then visualized with Western Lightning Plus-ECL (PerkinElmer, Waltham, MA, USA). The ratios of the band intensities were determined with the ImageJ software using X-ray films with non-saturated signals for the samples being compared.

### Chromatin fractionation and co-immunoprecipitation

Preparation of the chromatin-associated protein fractionation was performed as described previously [44]. Briefly, cells were harvested and lysed for 45 min on a rotator at 4°C in buffer A (50 mM Tris-HCl [pH 7.5], 1 mM DTT, and 0.5% Triton X-100, supplemented with 1× protease inhibitor cocktail containing no EDTA [Sigma-Aldrich, St. Louis, MO, USA]). After centrifugation at 1800 g at 4°C for 10 min, pellets were washed twice with buffer A, resuspended in buffer B (50 mM Tris-HCl [pH 8.0] and 1.5 mM CaCl_2_), and finally treated with 30 units of micrococcal nuclease (Takara Bio, Shiga, Japan) for 35 min at 37°C under mild agitation. Solubilized proteins were clarified by two rounds of centrifugation at 5000 g at 4°C for 2 min. Before immunoprecipitation, the chromatin fraction was adjusted to a final concentration of 150 mM sodium chloride and 0.5% Triton X-100. Equal amounts of solubilized chromatin were incubated with the appropriate primary antibody at 4°C overnight, followed by addition of 30 μl of Dynabeads M-280 Sheep Anti-Mouse/Rabbit IgG (Life Technologies, Carlsbad, CA, USA) pre-blocked with 5% BSA in IP buffer. The beads were washed four times with Tris-buffered saline (TBS) containing 0.1% Triton X-100 and 0.25% NP-40. Finally, purified proteins were eluted in Laemmli buffer and subjected to western blotting.

### *In vitro* kinase assay

Recombinant EZH2/EED/SUZ12/RbAp48/AEBP2 complex (BPS Bioscience, San Diego, CA, USA) (700 ng) was incubated with 100 ng of recombinant LATS2 or LATS1 kinases (Carna Biosciences, Hyogo, Japan) at 30°C for 30 min with kinase reaction buffer (5 mM MOPS-NaOH [pH 7.2], 5 mM magnesium chloride, 1 mM EGTA, 0.4 mM EDTA, 5 mM glycerol 2-phosphate, 50 μM DTT, and 50 μM ATP). For protein phosphatase (PPase) assay, 200 U of λ-PPase (New England Biolabs, Ipswich, MA, USA) was added to the kinase reaction tube. Each reaction was carried out in a 25 μl volume. The reaction was stopped by addition of 4× Laemmli sample buffer. Proteins were separated by SDS-PAGE in gels containing 50 μM Phos-tag acrylamide (WAKO, Osaka, Japan) and subjected to western blotting.

### *In vivo* kinase assay

To verify the effects of LATS2 kinase activity on the phosphorylation state of EZH2 in cells, FLAG-tagged human EZH2 was transiently overexpressed in each add-back cell line using Lipofectamine (Life Technologies, Carlsbad, CA, USA) and PLUS reagents (Life Technologies, Carlsbad, CA, USA). Whole-cell lysates were generated 48 h after transfection. Proteins were separated by SDS-PAGE in gels containing 50 μM Phos-tag acrylamide (WAKO, Osaka, Japan) and subjected to western blotting as described above.

### *In vitro* histone methyltransferase assay

Histone methyltransferase (HMTase) assay was performed using immunoprecipitated EZH2 and its co-precipitating proteins. Briefly, native chromatin from each sample was extracted as described for ChIP-qPCR below, without fixation. Appropriate amounts of solubilized chromatin were incubated with 2 μg of anti-EZH2 antibody (Active Motif, Carlsbad, CA, USA) and 20 μl of Dynabeads M-280 Sheep Anti-Mouse IgG (Life Technologies, Carlsbad, CA, USA) at 4°C for 4 h. The beads were washed two times with ChIP buffer (10 mM Tris-HCl [pH 8.0], 200 mM KCl, 1 mM CaCl_2_, and 0.5% NP-40), two times with Wash buffer (10 mM Tris-HCl [pH 8.0], 500 mM KCl, 1 mM CaCl_2_, and 0.5% NP-40), and once with HMTase buffer (20 mM phosphate buffer [pH 7.4] and 0.05% Tween-20). The immunoprecipitated protein was incubated at 30°C for 3 h in 30 μl of HMTase buffer containing 1 μg of recombinant histone H3.1 protein (New England Biolabs, Ipswich, MA, USA) as substrate and 40 μM S-adenosylmethionine (SAM) (New England Biolabs, Ipswich, MA, USA) as the methyl donor. The reaction was stopped by addition of 10 μL of 4× Laemmli sample buffer. Proteins were separated by SDS-PAGE and subjected to western blotting. Each western blotting signal was quantified using the ImageJ software as described above, and the H3K27me3 level was normalized to the immunoprecipitated EZH2 protein signal.

### Rescue of LATS2-KO by transient expression of LATS2 and/or EZH2

MYC-tagged LATS2 and/or FLAG-tagged EZH2 were transiently overexpressed in LATS2-KO HeLa-S3 cells using Lipofectamine (Life Technologies, Carlsbad, CA, USA) and PLUS reagents (Life Technologies, Carlsbad, CA, USA). To evaluate the synergetic effects and the dose dependency of LATS2, the amount of co-transfected LATS2-plasmid was continuously increased up to the amount used for LATS2 transfection alone. Whole-cell lysates were generated 48 h after transfection and analyzed by western blotting.

### Cell-cycle analysis

HeLa-S3 cells were synchronized by the double thymidine-block method and collected at various time points. A portion of the cells was fixed by incubating cells in cold 70% (w/v) ethanol at 4°C for 30 min with brief vortexing. The fixed cells were washed with PBS (-), treated with a propidium iodide solution containing RNase A, and sorted on a FACSCalibur flow cytometer (Becton Dickinson, Franklin Lakes, NJ, USA) using the CellQuest software. Total RNA and whole protein lysates were extracted from the remaining cells using QIAzol Lysis Reagent (Qiagen, Hilden, Germany) and the RNeasy Mini Kit (Qiagen, Hilden, Germany). Each sample was subjected to RT-qPCR analysis and western blotting.

### RT-qPCR

To quantitate expression of each gene, total RNA was extracted from cell cultures by direct lysis of cells on dishes using the QIAzol Lysis Reagent (Qiagen, Hilden, Germany), followed by RNA purification using RNeasy Mini Kits (Qiagen, Hilden, Germany). cDNAs were synthesized using the High-Capacity cDNA Reverse Transcription Kit (Life Technologies, Danvers, MA, USA). Quantitative PCR analysis was performed on a 7900HT Fast Real-Time PCR System (Applied Biosystems), using SYBR Premix Ex Taq II (Tli RNase H Plus) and Premix Ex Taq™ (Perfect Real Time) (Takara Bio, Shiga, Japan) for the SYBR Green method and TaqMan assays, respectively. Detailed sequences of the primer sets and the Assay IDs of the TaqMan assays used in this study are provided in the S7 and S8 Tables.

### Microarray analysis

Microarray analyses for coding genes and microRNAs were performed as single-color or two-color hybridizations using Agilent Whole Human/Mouse Genome Oligonucleotide Microarrays (Agilent Technologies, Santa Clara, CA, USA) as described in our previous work [45]. Agilent Feature Extraction software (v. 10.5.1) was used to assess spot quality and extract feature intensity statistics. The Subio Platform and Subio Basic Plug-in (v1.18) (Subio, Kagoshima, Japan) were used to calculate fold changes between samples. Briefly, to obtain the list of high-confidence expressing genes upon LATS2 knockdown, the spots with wellAboveBG-FLAG = TRUE in each sample group (i.e., probes that were distinguishable from the local background signal across samples) were selected. In addition, a minimum fold change of 1.4 was required for inclusion in the final list of differentially expressed genes. For LATS2-KO MEFs, a fold change ≥ 2.0 and p < 0.05 (t-test) was required for differentially expressed genes. The microarray data were deposited in the Gene Expression Omnibus (www.ncbi.nlm.nih.gov/geo) under accession number GSE63538.

### Library preparation and RNA-sequencing

Poly(A)+ RNA was isolated with Nucleo-Trap mRNA kit (Macherey-Nagel, Düren, Germany) and double strand cDNA synthesis was carried out using the double-stranded cDNA using SuperScript double-Stranded cDNA synthesis kit (Invitrogen, Carlsbad, CA, USA) according to manufacturer's instructions. Each double-stranded cDNA (120 ng) was sheared to ~400 bp fragments using an S220 ultrasonicator (Covaris, Woburn, MA, USA) with the following parameter settings: peak incident power, 140 W; duty factor, 10%; cycles per burst, 200; and treatment time, 55 seconds. The resulting DNA fragments were purified using 0.7× volume Agencourt AMPureXP beads (Beckman Coulter, Brea, CA, USA). Illumina libraries were prepared using the KAPA Library Preparation Kit (Kapa Biosystems, Wilmington, MA, USA) and TruSeq adaptors (Illumina, San Diego, CA, USA). Paired-end sequencing (151 bp × 2) of each sample was performed on a HiSeq2500 (Illumina, San Diego, CA, USA).

### RNA-seq data analysis

Raw images were processed using Real Time Analysis ver. 1.17.21 (Illumina, San Diego, CA, USA), and conversion to fastq file format was performed using CASAVA ver. 1.8.2 (Illumina, San Diego, CA, USA). Btrim (http://graphics.med.yale.edu/trim/readme) was used to trim low-quality regions of raw reads. The trimmed reads were mapped onto the reference human genome (hg19) using TopHat ver. 2.0.11 [46] in combination with Bowtie ver. 2.2.2 [47] and SAMtools ver. 0.1.19 [48]. Gene expression was quantitated with Cufflinks ver. 2.2.1 [49].

### ChIP-qPCR

Cells were cultured in 10 cm plates to approximately 80% confluence. Formaldehyde (Nacalai Tesque, Kyoto, Japan) was added directly to the culture medium to a final concentration of 0.5%. Crosslinking was allowed to proceed for 5 min at room temperature, and the formaldehyde was neutralized with glycine at a final concentration of 0.125 M for 5 min. After washing twice with ice-cold PBS, cells were collected, pelleted, resuspended in swelling buffer (25 mM HEPES [pH 7.8], 1.5 mM MgCl_2_, 10 mM KCl, 0.1% (w/v) Nonidet P-40, and 1 mM DTT, plus protease and phosphatase inhibitors), and incubated for 10 min on ice. Nuclei were released by subjecting the samples to 30 strokes in a Dounce homogenizer, collected, and resuspended in sonication buffer (50 mM HEPES [pH 7.9], 140 mM sodium chloride, 1 mM EDTA, 1% [w/v] Triton X-100, 0.1% [w/v] sodium deoxycholate, and 0.1% sodium dodecyl sulfate, plus protease and phosphatase inhibitors). Samples were sonicated in a Tomy UD-201 (TOMY SEIKO, Tokyo, Japan) for five cycles of 1 min each (50% duty, output level 2) separated by intervals of 1 min. Sonicated samples were clarified by spinning at 18,000 g at 4°C for 10 min. Equal amounts of sheared chromatin were incubated with the appropriate primary antibody at 4°C overnight, followed by addition of 30 μl of Dynabeads M-280 Sheep Anti-Mouse/Rabbit IgG (Life Technologies, Carlsbad, CA, USA) pre-blocked with 5% BSA in sonication buffer. The beads were washed twice each with sonication buffer, high-salt wash buffer (sonication buffer containing 500 mM sodium chloride), LiCl wash buffer (20 mM Tris [pH 8.0], 1 mM EDTA, 250 mM LiCl, 0.5% [w/v] Nonidet P-40, and 0.5% [w/v] sodium deoxycholate), and TE buffer (10 mM Tris [pH 8.0] and 1 mM EDTA). Immunoprecipitates were incubated at 65°C in elution buffer (50 mM Tris-HCl [pH 8.0], 10 mM EDTA, and 1% [w/v] sodium dodecyl sulfate) for 30 min, and then treated with 2 μg of Proteinase K (Sigma-Aldrich, St. Louis, MO, USA) overnight for de-crosslinking. Eluate was purified using the ChIP DNA Purification Kit (Active Motif, Carlsbad, CA, USA). For quantitation, ChIP DNA and input genomic DNA were subjected to qPCR on a 7900HT Fast Real-Time PCR System (Applied Biosystems, Waltham, MA, USA), using SYBR Premix Ex Taq II (Tli RNase H Plus) and Premix Ex Taq™ (Perfect Real Time) (Takara Bio, Shiga, Japan). Detailed sequences of the primer sets used are provided in the S7 Table.

### ChIP-sequencing

ChIP DNA and input DNA ends were repaired using T4 DNA polymerase, Klenow enzyme, and T4 polynucleotide kinase (PNK) (New England Biolabs, Ipswich, MA, USA), followed by treatment with Klenow exo- to add an A base to the 3’-end. After ligation of the Genomic Adaptor Oligo Mix (Illumina, San Diego, CA, USA) using TaKaRa Ligation Mix (Takara Bio, Shiga, Japan), the adaptor-ligated DNA fragments were amplified with Paired-End Sample Prep Oligo primers (Illumina, San Diego, CA, USA) for 18 cycles. The amplified library was separated on a 2.0% agarose gel, and the samples were purified using the QIAquick MinElute kit (Qiagen, Hilden, Germany) after each preparation step. The purified library was used for cluster generation and sequencing analysis on a HiSeq 2000 (Illumina, San Diego, CA, USA). The raw Illumina sequencing data are available from the Gene Expression Omnibus (www.ncbi.nlm.nih.gov/geo) under accession number GSE63538.

### ChIP-seq data analysis

Sequence reads for H3K27me3, H3K4me3, and input were aligned to the human genome (hg19) using the Bowtie software (parameter: -v 3 –m 1) [50]. The MACS software (ver. 1.4.1) was used for peak detection of each histone mark [27]. The parameters for MACS were ‘—nomodel—extsize 146—broad—to-large—pvalue 1e-3’, and the other parameters were the software defaults. Genes were called in association with a given chromatin mark only when peaks were called within ± 5 kb of the TSS. To calculate normalized depth around TSSs of all RefSeq genes, and to perform GO analysis of the called genes, the Homer software was used with the default settings [51]. P-values were corrected by the FDR (q-value) correction by R for multiple comparisons. To visualize normalized ChIP profiles in genome browser, BigWig files were generated using our custom scripts and visualized using the IGV software from the Broad Institute [51, 52]

### Immunofluorescence imaging

Exponentially growing HeLa-S3 cells were plated on coverslips and fixed for 15 min at room temperature in 4% formaldehyde in PBS, 0.1% Triton X-100 in PBS(-), and 0.05% Tween-20 in PBS. Fixed cells were rinsed three times in 1× PBS for 5 min each. To visualize H3K27me3 and RNAPII, cells were blocked in blocking buffer (1× PBS, 5% normal serum, and 0.3% Triton X-100) for 60 min, incubated with anti-H3K27me3 (Cell Signaling Technologies, Danvers, MA, USA) and anti-RNA polymerase II clone STD4H8 (Millipore, Billerica, MA, USA) antibodies, and then incubated with Alexa Fluor 488 and 594 (Molecular Probes, Eugene, OR, USA)-conjugated anti-rabbit/mouse IgG in 1× PBS containing 1% BSA and 0.3% Triton X-100. DNA was stained using Hoechst 33258 (Sigma-Aldrich, St. Louis, MO, USA), and cells were observed on a FluoView FV10i microscope (Olympus, Tokyo, Japan).

### Statistical analysis by NextBio

For meta-analysis and exploration of massive preprocessed omics data (reported in previous studies) that exhibited significant correlation with our own data, each processed omics dataset was uploaded into the NextBio enterprise software (Illumina, San Diego, CA, USA), and the statistical significance of the relationships was evaluated as reported previously [28]. For Canonical pathway enrichment analysis, p-values were subjected to FDR (q-value) correction in the R statistical computing environment.

### GSEA

To determine whether gene sets of interest were statistically enriched among up- and downregulated genes, we analyzed our non-redundant list of genes using GSEA 2.0 for pre-ranked lists [25]. The gene sets used in this study (e.g. ‘C2 cgp collection’) were obtained from the Broad Molecular Signatures Database (MSigDB).

### Promoter classification

To classify human coding genes by the CG status of their promoters, human coding gene IDs were obtained from Ensembl database. Genes >3 kb in length, with no other genes within 500 bp of their TSSs, were used for the promoter analysis. A BED format file of the filtered genes' promoters (from ˗1200 bp to +300 bp relative to the TSS) was generated, and each promoter region was divided into 500 bp sliding windows (5 bp offset), and the CpG ratio and CG% were calculated using Bedtools [53]. Next, each promoter was classified into one of three types according to the criteria described in previous studies [54]. The resultant lists of genes were uploaded into the NextBio platform and subjected to successive statistical analyses. For statistical analysis of RNA-seq data, fold changes were calculated for each actively transcribed gene, and then the Wilcoxon rank-sum test was performed to evaluate statistical significance.

### Analysis of TCGA data

To visualize expression patterns of LATS1 and LATS2 genes in many types of human cancers, PANCAN normalized RNA-seq data from the TCGA project were downloaded from the Cancer Browser website. Cancer datasets with at least one normal solid tissue sample were visualized as box-and-whisker plots. For analysis of glioblastoma multiforme (GBM) samples, level 3 preprocessed expression data from Agilent 244K custom gene expression G4502A_07_2 microarrays of 483 clinical samples, along with the corresponding clinical data, were downloaded from the TCGA Data Portal. Data were visualized as box-and-whisker plots for each sample group, and the Wilcoxon rank-sum test was performed to evaluate statistical significance; Kaplan–Meier survival analysis followed by a log-rank test was performed using the ‘survival’ package in R. For GSEA analysis of the aggregated expression profile based on LATS2 expression level, a non-redundant list of genes was generated based on the mean fold change, and then GSEA for pre-ranked lists was performed as described above.

### Ingenuity Pathways Analysis

The ‘Core Analysis’ function included in IPA software (Qiagen, Hilden, Germany) was used to examine the microarray data of *Lats2* KO MEFs in the differentiation processes. All DEGs of the three microarray experiments were subjected to IPA software with default setting, then significance of the canonical pathways related to differentiation processes were visualized in heatmaps according to calculated z-value.

## Supporting Information

S1 File Supporting Information. (PDF)

S1 Table Differentially expressed genes in *LATS2* KO HeLa-S3 cells. (XLSX)

S2 Table Differentially expressed genes in HeLa-S3 cells upon LATS2 knockdown. (XLSX)

S3 Table Top 25 gene sets positively enriched in *LATS2* KO HeLa-S3 cells (XLSX)

S4 Table LATS2-dependent H3K27me3 targets in *LATS2* KO HeLa-S3 cells. (XLSX)

S5 Table Differentially expressed genes in *Lats2* KO MEFs. (XLSX)

S6 Table Differentially Expressed Genes of MEFs in a Hippo-inactive state. (XLSX)

S7 Table Primer sequences used in this study. (XLSX)

S8 Table TaqMan probes used in this study. (XLSX)

S9 Table Antibodies used in this study. (XLSX)
