# Supplementary material for: LATS2 Positively Regulates Polycomb Repressive Complex 2
Source: PLoS One. 2016 Jul 19;11(7):e0158562. doi: 10.1371/journal.pone.0158562 (PMC4951031; doi:10.1371/journal.pone.0158562)
Supplement: S1 File — (PDF) [file pone.0158562.s001.pdf]

S1 File. Supporting Information

LATS2 kinase positively regulates Polycomb repressive complex 2

Kosuke Torigata, Daisuke Okuzaki, Satomi Mukai, Akira Hatanaka, Fumiharu Ohka, Daisuke Motooka, Shota Nakamura, Yasuyuki Ohkawa, Norikazu Yabuta, Yutaka Kondo, Hiroshi Nojima*

* To whom correspondence should be addressed.

S1 File. Supporting Information includes:

Supplementary Results and Discussion for Fig C–J

Supplementary References

Supplementary Figures A–J and Legends

Supplementary Results and Discussion for Fig C–J

Tissue or context specificity of LATS2-dependent H3K27me3 targets

We attempted to explore the potential physiological relevance of the LATS2-responsive PRC2 signal. To more precisely evaluate the LATS2-dependent epigenetic signature, we analyzed ChIP-seq and RNA-seq data based on properties of their promoters. To this end, we categorized promoters of protein-coding genes into three classes based on their CpG content [1]: 1) High-CpG promoter (HCP), associated with both housekeeping genes and other genes with complicated expression patterns during development. This class often includes genes with bivalent states, i.e., those that possess both H3K27me3 and H3K4me3 marks in their promoters; 2) Low-CpG promoter (LCP), associated with genes that are under control of tissue-specific transcription factors and are often repressed by DNA methylation. This class exhibits tissue-specific expression pattern during differentiation; and 3) Intermediate-CpG promoter (ICP), i.e., genes which are neither HCPs nor LCPs. (HCP, LCP and ICP) (Fig **Ca**). Promoter analysis of potential LATS2-dependent epigenetic signatures, i.e., ‘H3K27me3-loss’ module from HeLa-S3 study, revealed that this module significantly correlated with the LCP class ($p = 5.2E-117$) (Fig **Cb**). Consistent with this, fluctuations in gene expression were larger in the LCP class than in HCP or ICP (Fig **Cc**). LCP genes are associated with tissue-specific transcription programs during differentiation. Indeed, the LATS2-responsive PRC2 target genes in HeLa-S3 cells correlated with genes harboring the H3K27me3 mark specifically in human ES cells (vs. human fibroblasts, used as a reference sample) ($p = 2.0E-173$) (Fig **Cd**).

Next, to reveal the tissue or context specificity of LATS2-dependent H3K27me3, we explored tissue-specific expression patterns of ‘H3K27me3-loss’ genes using Body Atlas tools of NextBio statistical platform. Intriguingly, LATS2-dependent H3K27me3 target genes were expressed at relatively low levels in embryonic cells, but were over-expressed in the nervous system (Fig **Da** and **Db**). These observations indicate that the expression pattern of the LATS2–PRC2 signal is converted to an active state during neurogenesis. Indeed, the expression profile of *LATS2* KO HeLa-S3 cells (relative to wild type) positively correlated with that of differentiated neurons relative to neural stem cells (Fig **Dc** and **S5** Table), i.e., the expression profile of cells with LATS2 correlated with that of neural stem cells. Further analysis revealed that the expression of genes with LATS2-dependent H3K27me3 marks in HeLa-S3 was highly induced during the differentiation of ES cells into mature neuron cells ($p < 0.001$) (Fig **Dd**). The tissue-

specific induction of 'H3K27me3-loss' genes in neural differentiation process was further supported by another GSEA using previous microarray studies for various *in vitro* differentiation systems: significant positive enrichment of 'H3K27me3-loss' genes was observed in only the neural differentiation experiment (one of six differentiation systems, Fig **E**). These results suggest that potential LATS2-responsive H3K27me3 targets are repressed in dedifferentiated cells, and then gradually induced in the proneural stage.

Association between LATS2 and PRC2 in brain tumorigenesis

Because Lats2 may contribute (via PRC2) to maintenance of the dedifferentiated state in the nervous system, we hypothesized that LATS2 can contribute to tumorigenesis in neural cells. First, to characterize the expression of LATS2 in various types of cancer, we visualized the relative expression level of the LATS2 gene using 8,415 RNA-seq datasets from TCGA. In many types of cancer, LATS2 tended to be expressed at low levels in tumor samples relative to normal solid tissue, consistent with the known tumor-suppressive properties of LATS2. By contrast, in GBM, LATS2 was expressed at higher levels in tumor samples than in normal tissue (Fig **Fa**, upper panel). To characterize the expression of LATS2 in detail, we examined array-based transcriptome data from 583 samples, including ten normal brain samples, from the TCGA portal site. In this dataset, we again observed significantly higher LATS2 expression in primary tumor samples (Fig **Fb,** left panel). Common GBM patients can be divided into four expression subtypes [2]; LATS2 expression was elevated in all but the proneural subtype (Fig **Fc**), which has a transcription signature related to neurogenesis and exhibits a more differentiated expression pattern than the other subtypes. Intriguingly, LATS1 did not exhibit a similar expression pattern in GBM (Fig **Fa**, lower panel and **Fb**, right panel), suggesting that up-regulation of LATS2 is not simply a result of activation of the canonical Hippo signal cascade, in which LATS2 and LATS1 are functionally complementary to each other. These results are consistent with our model in which LATS2 expression contributes to dedifferentiation of neural cells.

We next performed GSEA to determine whether genes potentially targeted by H3K27me3 or genes related to neurogenesis are differentially expressed in LATS2-low GBM (i.e., whether positive effects of LATS2 on PRC2-mediated repression of genes for neural differentiation were disturbed). To obtain gene expression profiles that depend on LATS2, we first divided GBM cases into two groups: those with higher expression of LATS2 than the median for normal brain (n = 411, 'LATS2-high') and

those with lower-than-median expression (n = 54, 'LATS2-low'). We performed GSEA using this profile. Consistent with our hypothesis, known H3K27me3 targets were highly expressed in LATS2-low GBM samples (Fig **Ga**). Moreover, some neuron-specific transcripts were positively associated with LATS2-low GBM samples (Fig **Gb**), whereas the stem-cell signature was enriched in LATS2-high GBM samples (Fig **Gc**). Furthermore, we discovered an association between LATS2 expression and clinical prognosis. Kaplan–Meier curves and estimates of survival data revealed that patients with higher-than-median LATS2 expression exhibit poorer clinical prognoses than patients with lower-than-median LATS2 expression (n = 466; p = 0.00512) (Fig **Gd**). Importantly, the difference in the survival probability simply reflects the difference between the proneural subtype and the other subtypes. We observed a similar tendency in survival probability in an analysis using only proneural samples (n = 115) (Fig **Ge**). These results support the impact of the LATS2 signal via PRC2 in both development and dedifferentiation of the nervous system.

Transcriptome analysis of *Lats2* KO MEFs

Based on the observation and correlation of LATS2 with PRC2 related signals in nervous system, we next examined if LATS2 plays fundamental roles through PRC2 in this tissue. Indeed, *Lats2*-deficient mice exhibit embryonic lethality due to a defect in development of the central nervous system [3]. The insight of the association of Lats2 with PRC2 during neurogenesis is further supported by a previous study in *Drosophila*: a mutant of *Wts*, the *Drosophila* homolog of *Lats1/2*, phenocopies the effect of Polycomb group (PcG) mutants on dendrite maintenance [4]. To examine the functional link between Lats2 and PRC2 in mice, we next performed transcriptome analysis of *Lats2* KO MEFs (Fig **Ha** and **Hb**). GSEA revealed that potential PRC2-regulated genes were elevated in *Lats2* KO MEFs (p-value < 0.001) (Fig **Hc** and **Hd**). Furthermore, the up-regulated genes in *Lats2* KO MEFs were significantly correlated with those of up-regulated genes in *Eed* KO ES cells during differentiation (Fig **He**). These results suggest that Lats2 depletion in MEFs causes dysregulation of PRC2 function.

We further investigated whether the dysregulation of repressive epigenetic mechanisms caused by *Lats2* KO is related to differentiation processes or maintenance of stemness. We performed GSEA on the expression profile of *Lats2* KO MEFs, using signatures of differentiation or stemness defined in a previous meta-analysis of human ES cells and differentiating stem cells [5]. In agreement with the study, *Lats2* KO was associated with a significant enrichment of genes that are expressed during developmental

processes (Fig **Ia** and **Ib**). This result suggests that Lats2 may repress genes involved in development (via PRC2). Indeed, some well-known transcripts associated with differentiation, such as those of homeotic genes, were up-regulated in *Lats2* KO MEFs (Fig **Id** and **Ie**).

The canonical Hippo pathway contributes to various processes related to both development and dedifferentiation. The significant overlap of up-regulated genes in *Lats2* KO MEFs with genes for differentiation that we observed Figure S8, may be simply a result of canonical Hippo–YAP/TAZ signaling, which is caused by activation of Yap/Taz due to Lats2 depletion (a schematic; Fig **Ja**). To determine whether the *Lats2* KO signature was Yap/Taz-dependent, we compared gene expression in *Lats2* KO MEFs and rapidly growing wild type MEFs in log-phase, which have active Yap/Taz (validated in Fig **Jb**). Gene Ontology (GO) analysis of ontological terms related to differentiation processes revealed that 'Hippo-OFF' cells, i.e., growing cells, in which the intrinsic Hippo pathway is turned off and Yap/Taz is active, show associations with differentiation processes less significantly than *Lats2* KO cells. (Fig **Jc**, **S6** and **S7** Table). This result indicates that the association of up-regulated genes in *Lats2* KO MEFs with differentiation does not simply reflect the output of the canonical Hippo pathway.

In summary, a series of comparative analyses of LATS2-responsive H3K27me3 targets, cancer genomics data and *Lats2* KO MEFs present a possibility of an attractive novel function of LATS2 kinase in specific tissues and cellular contexts, especially neurogenesis, which will be clarified in our future research.

Supplementary References

1. Mikkelsen TS, Ku M, Jaffe DB, Issac B, Lieberman E, Giannoukos G, et al. Genome-wide maps of chromatin state in pluripotent and lineage-committed cells. Nature. 2007;448(7153):553-60. doi: 10.1038/nature06008. PubMed PMID: 17603471; PubMed Central PMCID: PMCPMC2921165.

2. The Cancer Genome Atlas Research Network.. Comprehensive genomic characterization defines human glioblastoma genes and core pathways. Nature. 2008;455(7216):1061-8. doi: 10.1038/nature07385. PubMed PMID: 18772890; PubMed Central PMCID: PMCPMC2671642.

3. Yabuta N, Okada N, Ito A, Hosomi T, Nishihara S, Sasayama Y, et al. Lats2 is an essential mitotic regulator required for the coordination of cell division. J Biol Chem. 2007;282(26):19259-71. doi: 10.1074/jbc.M608562200. PubMed PMID: 17478426.

4. Parrish JZ, Emoto K, Jan LY, Jan YN. Polycomb genes interact with the tumor suppressor genes hippo and warts in the maintenance of Drosophila sensory neuron dendrites. Genes Dev. 2007;21(8):956-72. doi: 10.1101/gad.1514507. PubMed PMID: 17437999; PubMed Central PMCID: PMCPMC1847713.

5. Assou S, Le Carrour T, Tondeur S, Ström S, Gabelle A, Marty S, et al. A meta-analysis of human embryonic stem cells transcriptome integrated into a web-based expression atlas. Stem Cells. 2007;25(4):961-73. doi: 10.1634/stemcells.2006-0352. PubMed PMID: 17204602; PubMed Central PMCID: PMCPMC1906587.

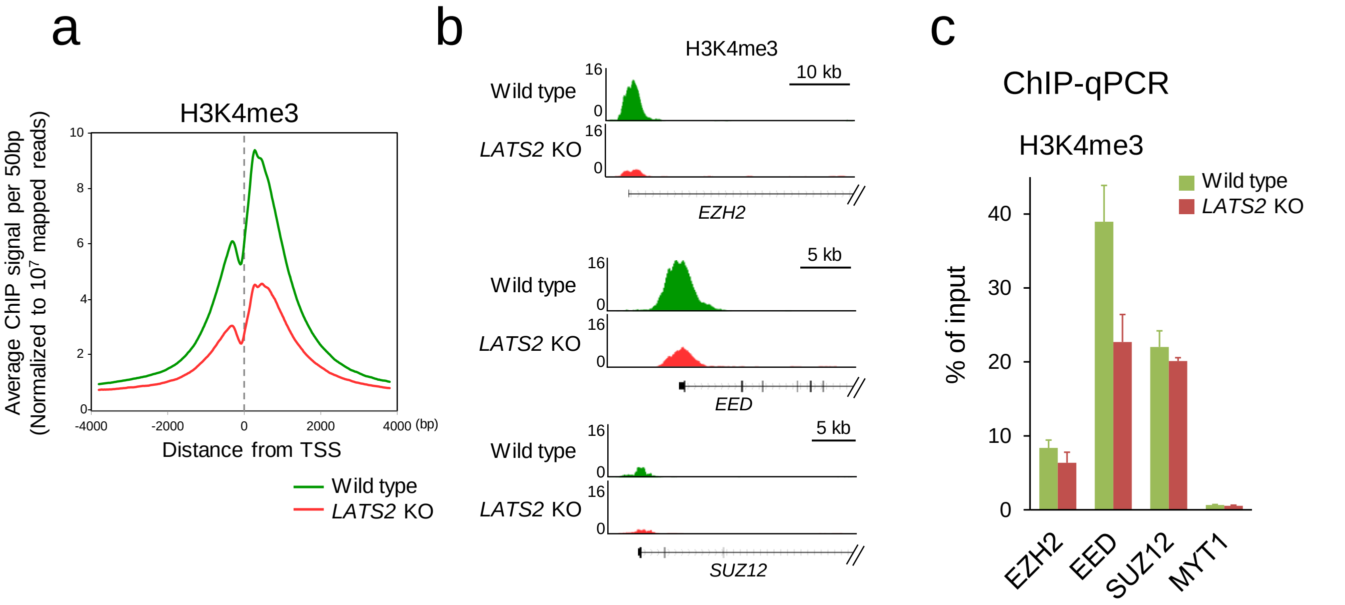

Supplementary Figure A. Characterization of H3K4me3 for PRC2 expression in HeLa-S3 cells. (**a**) Aggregate plots of H3K4me3 ChIP-seq signals centered at TSSs of all RefSeq genes in parental wild type (green) and *LATS2* KO HeLa-S3 cells (red). (**b**) Snapshots of ChIP-seq traces for H3K4me3 in wild type (green) and *LATS2* KO HeLa-S3 cells (red) at loci encoding the PRC2 core components. (**c**) ChIP-qPCR analysis for H3K4me3 at TSSs of genes encoding the core components of PRC2. The analysis reveals the loss of the H3K4me3 mark at the TSSs of EZH2 and EED, which are downregulated by *LATS2* knockout. The *MYT1* locus is a negative control region for H3K4me3, an active histone mark. All ChIP experiments were performed at least twice independently; error bars show SD.

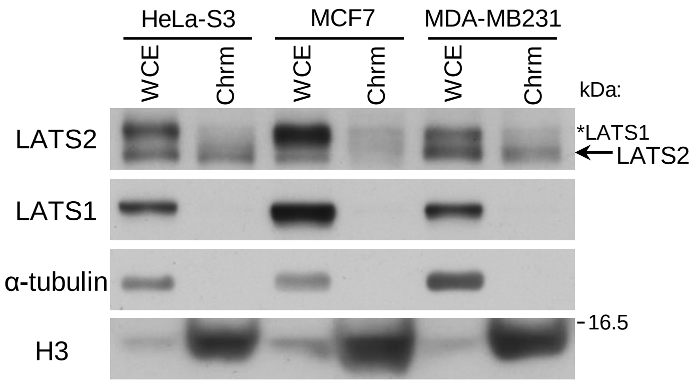

Supplementary Figure B. LATS2 localizes on chromatin in human cancer cell lines. Western blot analysis of LATS2 in some human cancer cell lines**.** α-tubulin and H3 were used to confirm successful fractionation. Arrow represents Lats2 signals. '*Lats1' represents Lats1 signals. WCE, whole cell extract. Chrm, solubilized chromatin fraction.

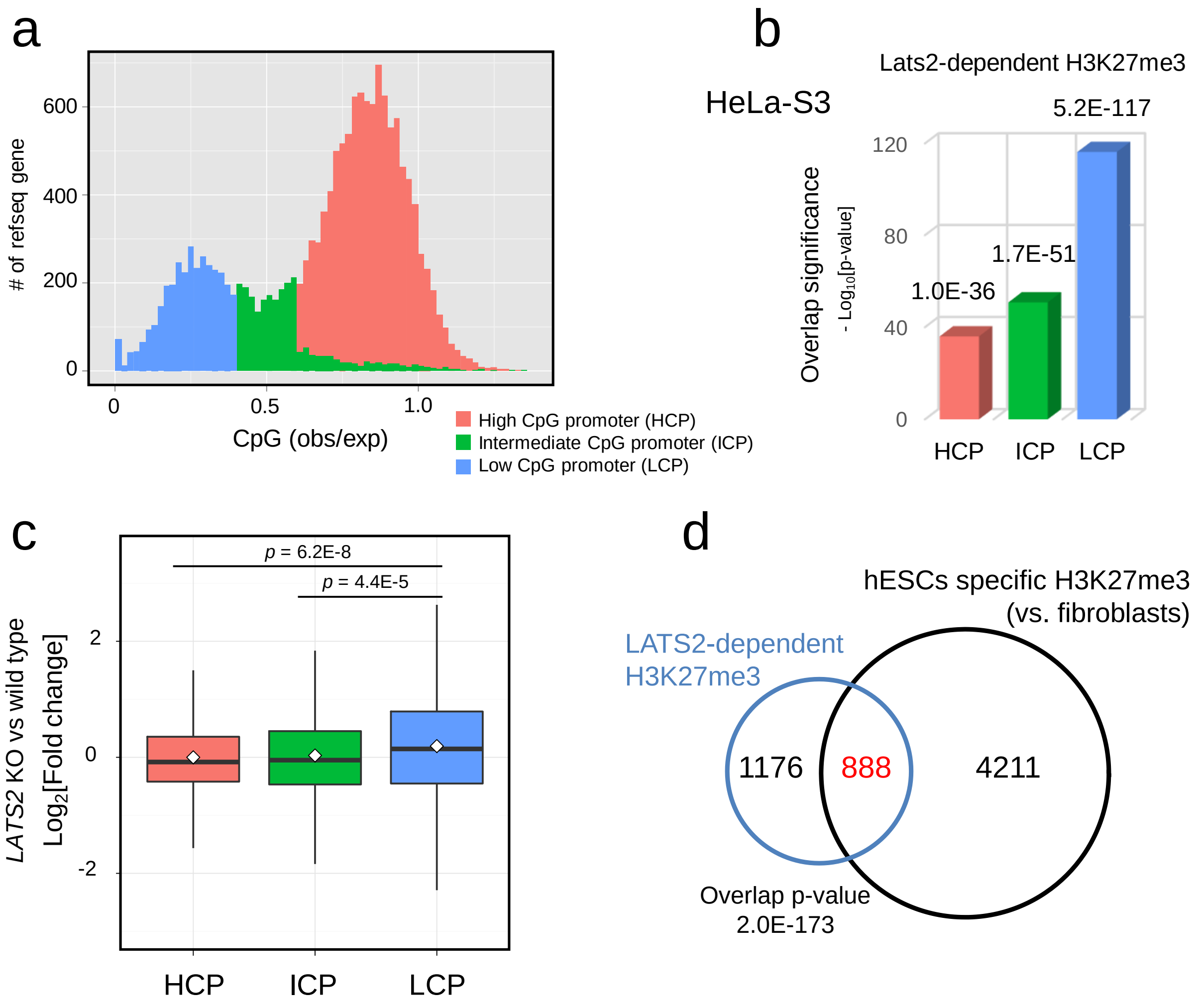

Supplementary Figure C. *LATS2* KO HeLa-S3 cells exhibit significant activation of LCPs. (**a**) Histogram showing the distribution of observed versus expected CpG frequencies for all promoters of protein-coding genes. The histogram shows a bimodal distribution of CpG-rich and CpG-poor promoters. All promoters were classified into three non-redundant three groups based on their CpG content as described previously [1]. (**b**) Bar plots showing the significance of overlap of genes with each class of promoters with genes containing LATS2-dependent H3K27me3 marks. (**c**) Box-and-whisker plots showing the expression level of transcriptionally active genes with different promoter classes, based on RNA-seq experiments. p-value was calculated by the Wilcoxon rank-sum test for comparison of two unpaired groups. In *LATS2* KO HeLa-S3 cells, significant genomic perturbations were observed in LCP promoters. (**d**) Venn diagram showing the overlap of genes in which H3K27me3 peaks within ±5 kb of the TSS decreased after *LATS2* KO in HeLa-S3 cells (blue) with genes harboring H3K27me3 marks specifically in human ESCs (i.e., genes in which the H3K27me3 level was significantly higher in hESCs than in human fibroblasts) (black). The 'H3K27me3-loss' genes from ChIP-seq experiment were subjected to NextBio analysis as LATS2-dependent H3K27me3 targets.

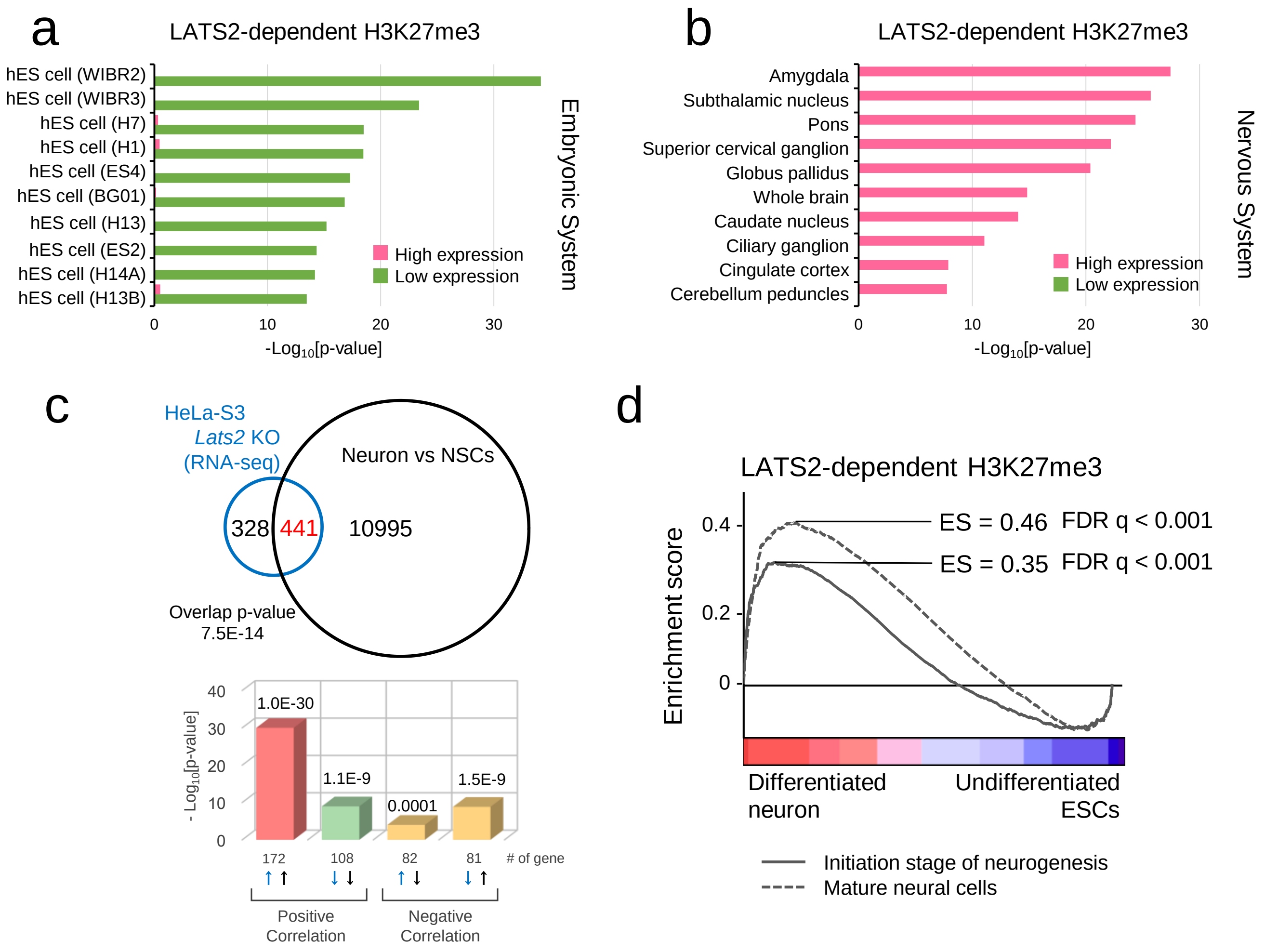

Supplementary Figure D. LATS2-dependent H3K27me3 targets are highly induced during neurogenesis. (**a**–**b**) The 'H3K27me3-loss' module exhibits an inverse expression pattern between embryonic cells and the nervous system. The 'H3K27me3-loss' genes were subjected to NextBio analysis as LATS2-dependent H3K27me3 targets, and then the tissue-specific expression pattern was extracted from the Body Atlas tool. (**c**) Significant overlap of DEGs in *LATS2* KO HeLa-S3 cells with genes involved in neurogenesis. DEGs in *LATS2* KO HeLa-S3 cells (≥2-fold, p-value <0.05) were subjected to NextBio analysis. Venn diagrams show the number of common and unique genes in both sets. Bar plots show the significance of overlap in each direction. (**d**) GSEA for the 'H3K27me3-loss' genes in human ES cells after induction of neural differentiation. Genes are ranked according to the fold change, derived from microarray experiments (differentiating cells vs. ES cells) (GSE28633). A positive enrichment score indicates increased expression after the induction of neural differentiation.

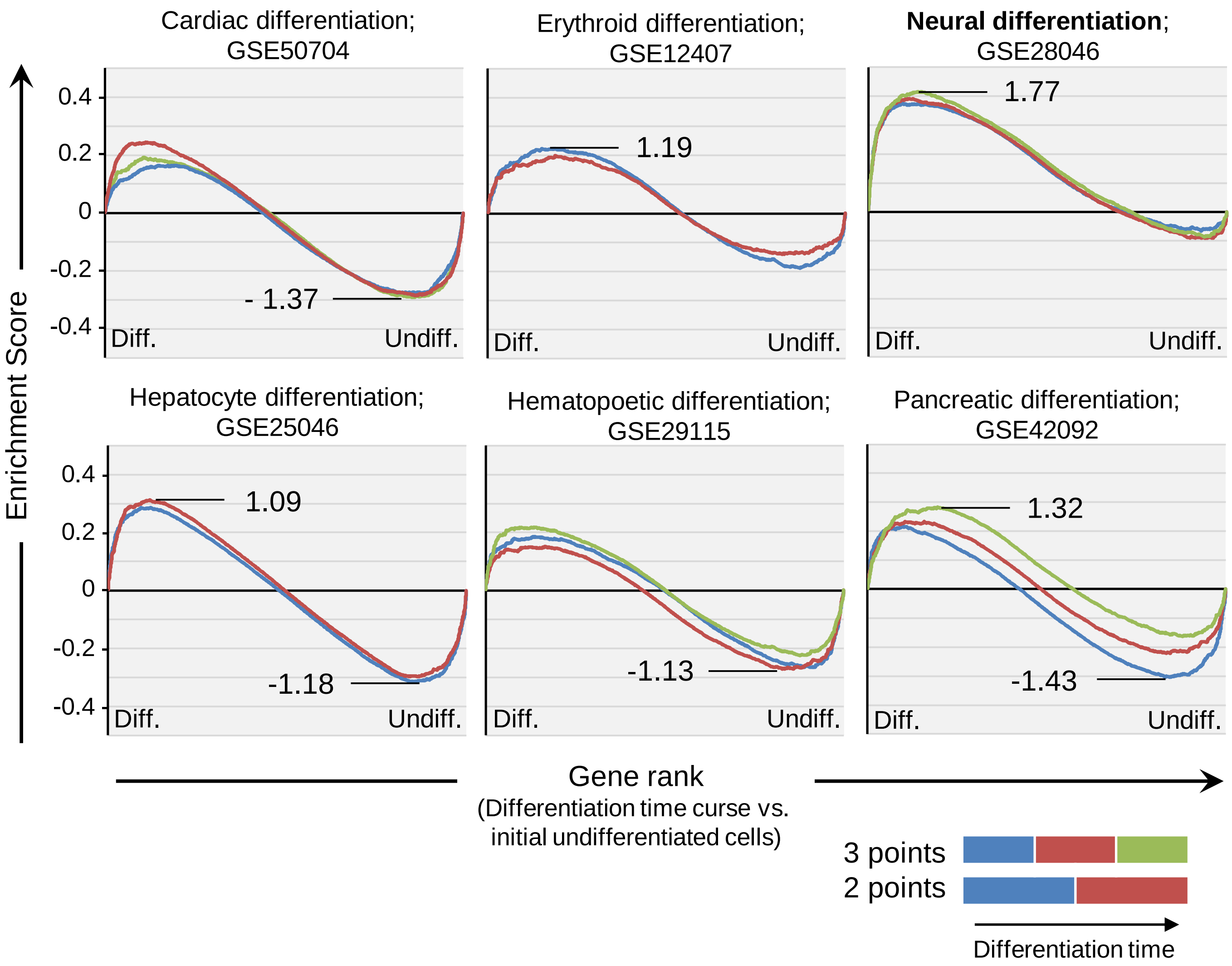

Supplementary Figure E. GSEA showing neural specific induction of LATS2-dependent H3K27me3 targets. GSEA for 'H3K27me3-loss' genes in various differentiation systems. Previous time-course studies of differentiation induction from un-differentiated cells were re-analyzed. Genes are ranked according to the fold change, derived from each microarray experiments (i.e., [each time point]/[un-differentiated samples]). A positive enrichment score indicates increased expression of LATS2-PRC2 targets after the induction of differentiation. Normalized enrichment scores were represented in the each panel.

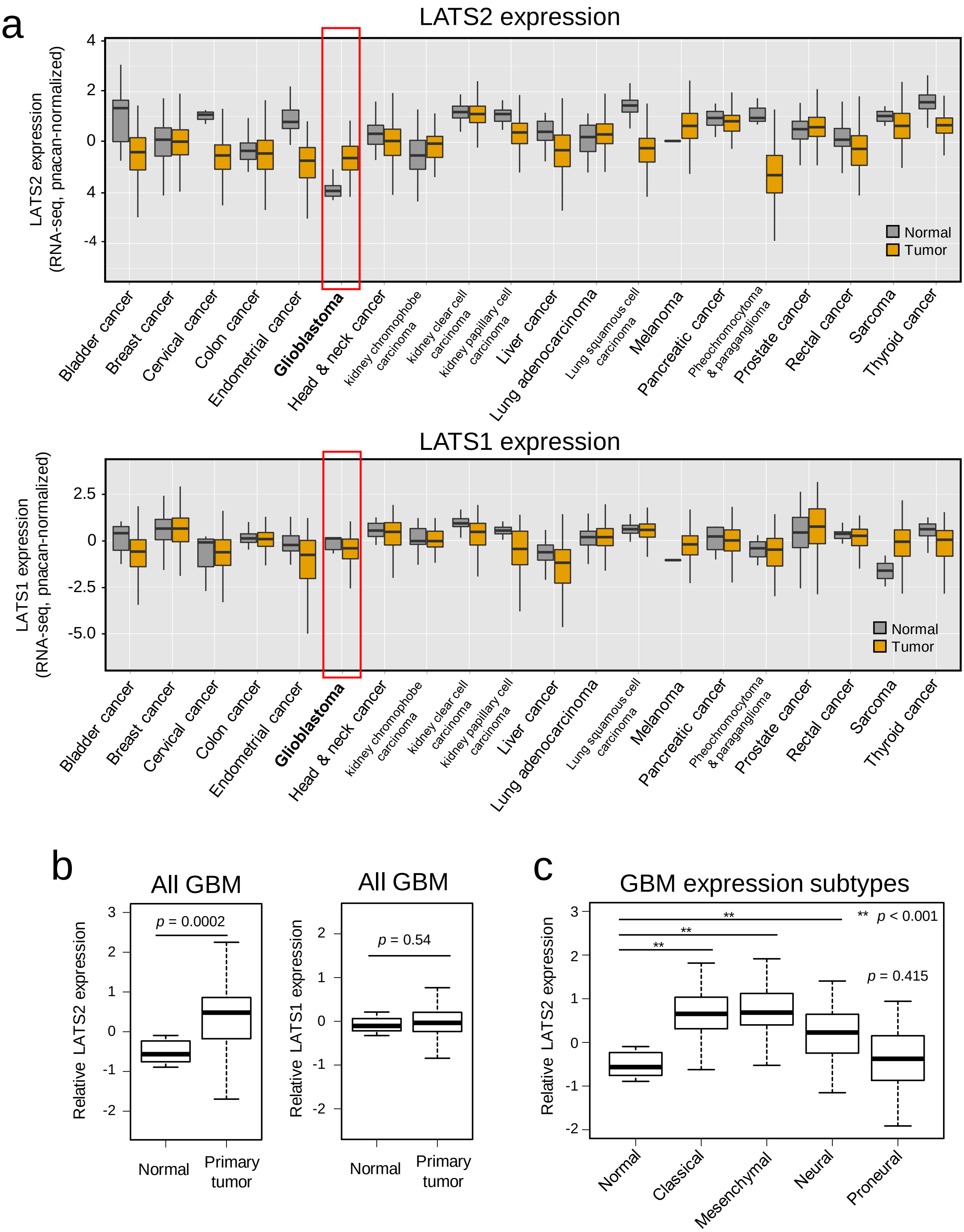

Supplementary Figure F. Not LATS1 but LATS2 shows enhanced expression in glioblastoma multiforme (GBM). (**a**) Box-and-whisker plots of mRNA expression of LATS2 (top) and LATS1 (bottom) in normal tissues and human cancers. mRNA expression data of LATS2 and LATS1 for various cancers and normal tissue samples were obtained from TCGA pan-cancer cohorts. Cancers with at least one normal sample were analyzed and visualized. (**b**) Box-and-whisker plots of LATS2 (left) and LATS1 (right) mRNA expression in normal brain and primary GBM. LATS2 mRNA expression data for GBM and normal tissue samples were obtained from level 3 preprocessed expression data from Agilent 244K custom gene-expression G4502A_07_2 microarrays. Statistical significance of differences between normal brain and primary tumor was evaluated by Wilcoxon rank-sum test. (**c**) Box-and-whisker plots of LATS2 mRNA expression in normal brain and predefined gene-expression subtypes of GBM. Statistical significance between normal brain and each subtype was evaluated by paired Wilcoxon rank-sum test.

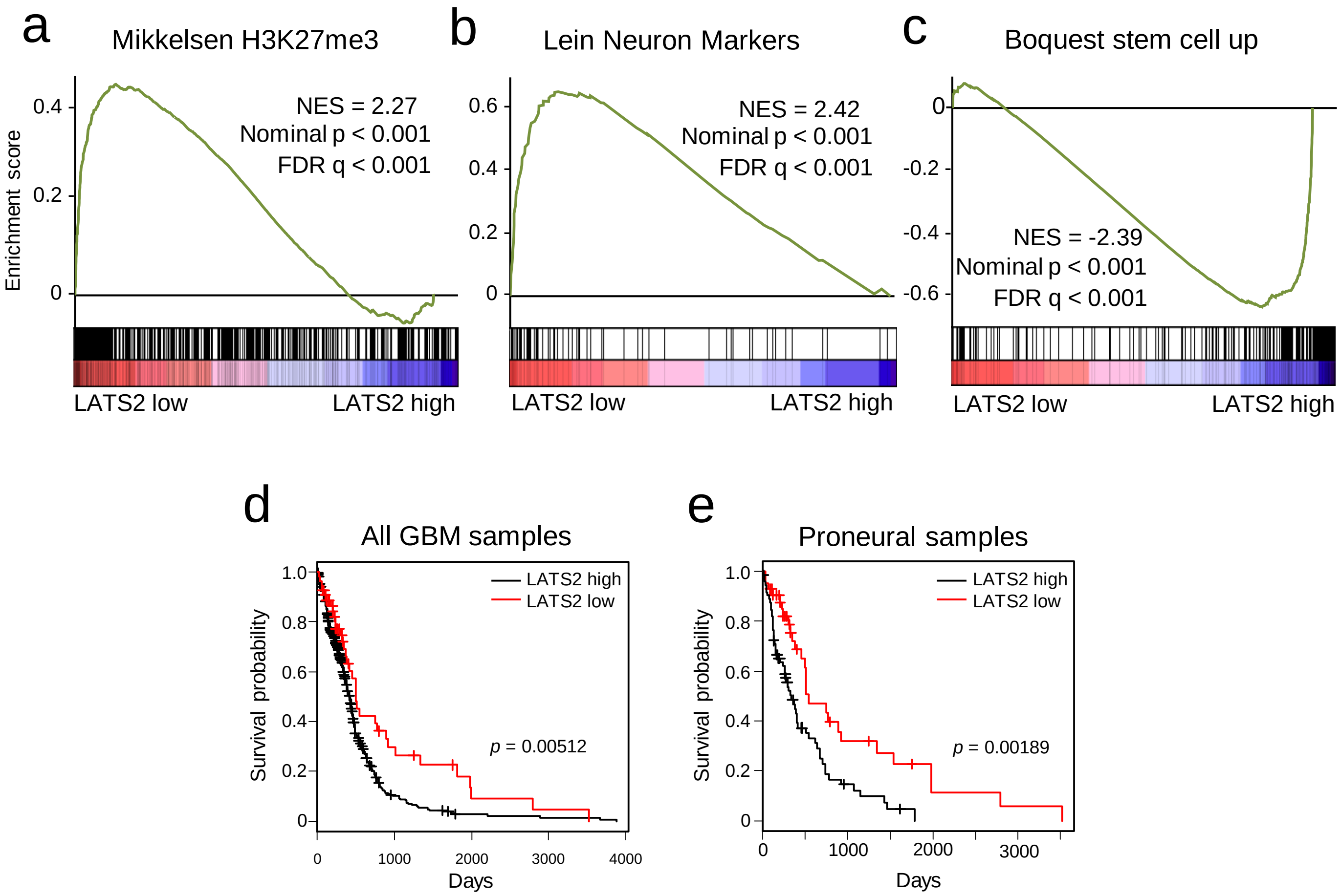

Supplementary Figure G. Expression level of LATS2 associates with epigenetic dysregulation and poor prognosis of GBM. (**a**–**c**) GSEA of GBM samples divided into two groups based on LATS2 level, for H3K27me3 targets (a), neuron marker genes (b), and stemness marker genes (c), respectively. Genes are ranked according to the average fold change derived from level 3 preprocessed expression data from Agilent 244K custom gene-expression G4502A_07_2 microarrays (samples with low LATS2 expression vs. samples with high LATS2 expression). A positive enrichment score indicates increased expression in LATS2-low GBM samples, and negative enrichment score indicates increased expression in LATS2-high GBM samples. (**d**) Kaplan–Meier survival curves for survival (days) of all GBM patients, stratified by LATS2 mRNA expression level. GBM patients were divided into two groups based on the median of normal samples. Statistical significance was assessed by log-rank test. (**e**) Kaplan–Meier survival curves for survival (days) of proneural-subtype patients, stratified by LATS2 mRNA expression level. Proneural-subtype samples were divided into two groups based on the median of normal samples. Statistical significance was assessed by a log-rank test.

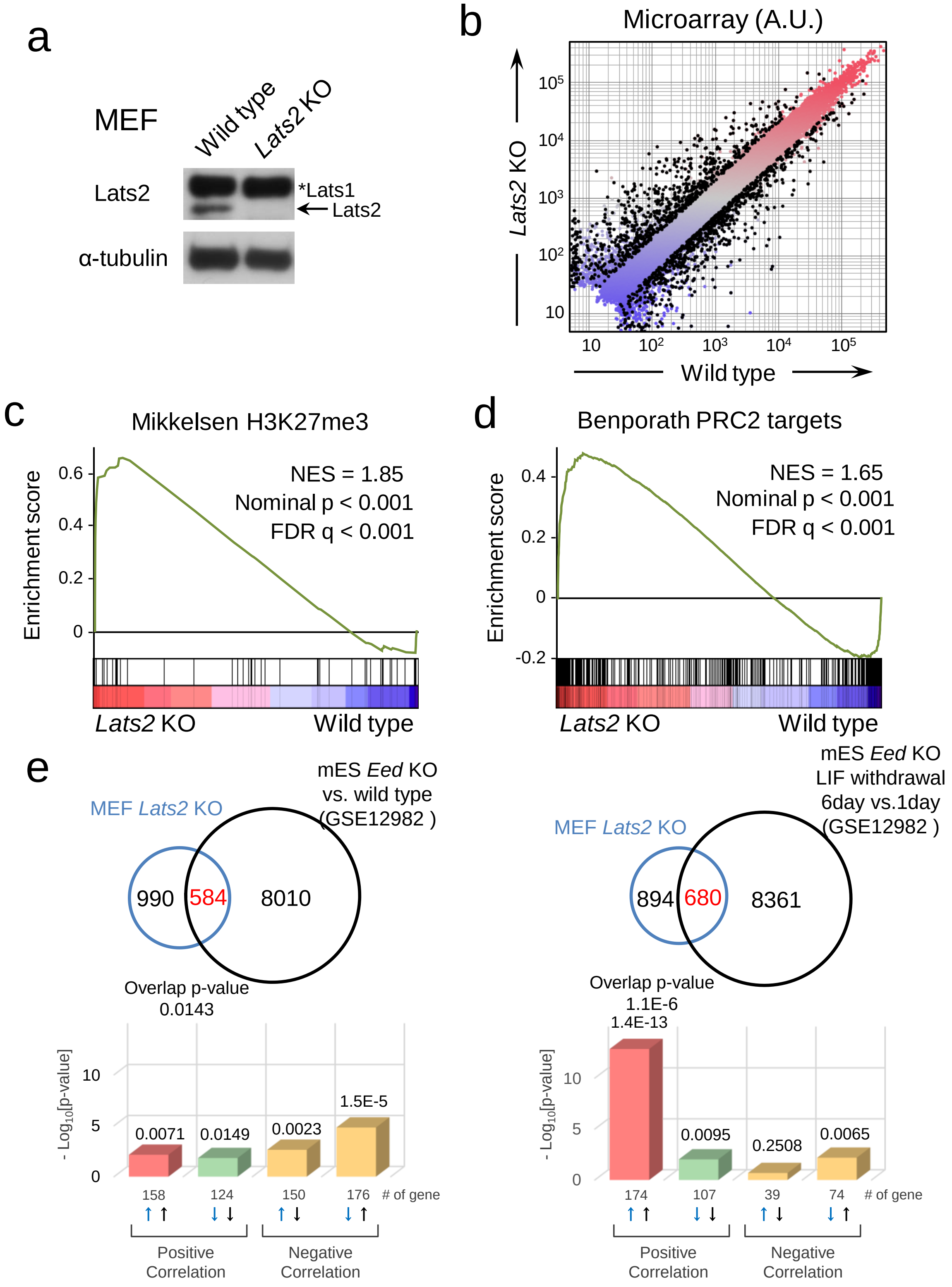

Supplementary Figure H. *Lats2* KO MEFs exhibit de-repression of PRC2 targets. (**a**) Confirmation of *Lats2* KO by western blotting. The anti-Lats2 polyclonal antibody used here recognizes the N-termini of both Lats2 and Lats1. The arrow represents Lats2 signals. '*Lats1' represents Lats1 signals. (**b**) Scatter plot of microarray data comparing *Lats2* KO and wild type MEFs. DEGs (≥2-fold, p-value <0.05) are highlighted by black dots. See also Supplementary Table S5 for more details. (**c**–**d**) GSEA of *Lats2*-KO MEFs for known H3K27me3-marked genes (c) and PRC2 targets (d). Genes ranked according to fold change (KO vs. WT littermate control). A positive enrichment score indicates higher expression after *Lats2* knockout. (**e**) Significant overlap of DEGs in *Lats2* KO MEFs with those induced during differentiation in *Eed* KO ES cells. DEGs in Lats2-KO MEFs (≥2-fold, p-value <0.05) were subjected to NextBio analysis. Venn diagrams show the number of common and unique genes in both sets. Bar plots show the significance of overlap in each direction. The result shows up-regulated genes in *Lats2* KO MEFs correlates with *Eed* target genes especially those regulated during differentiation.

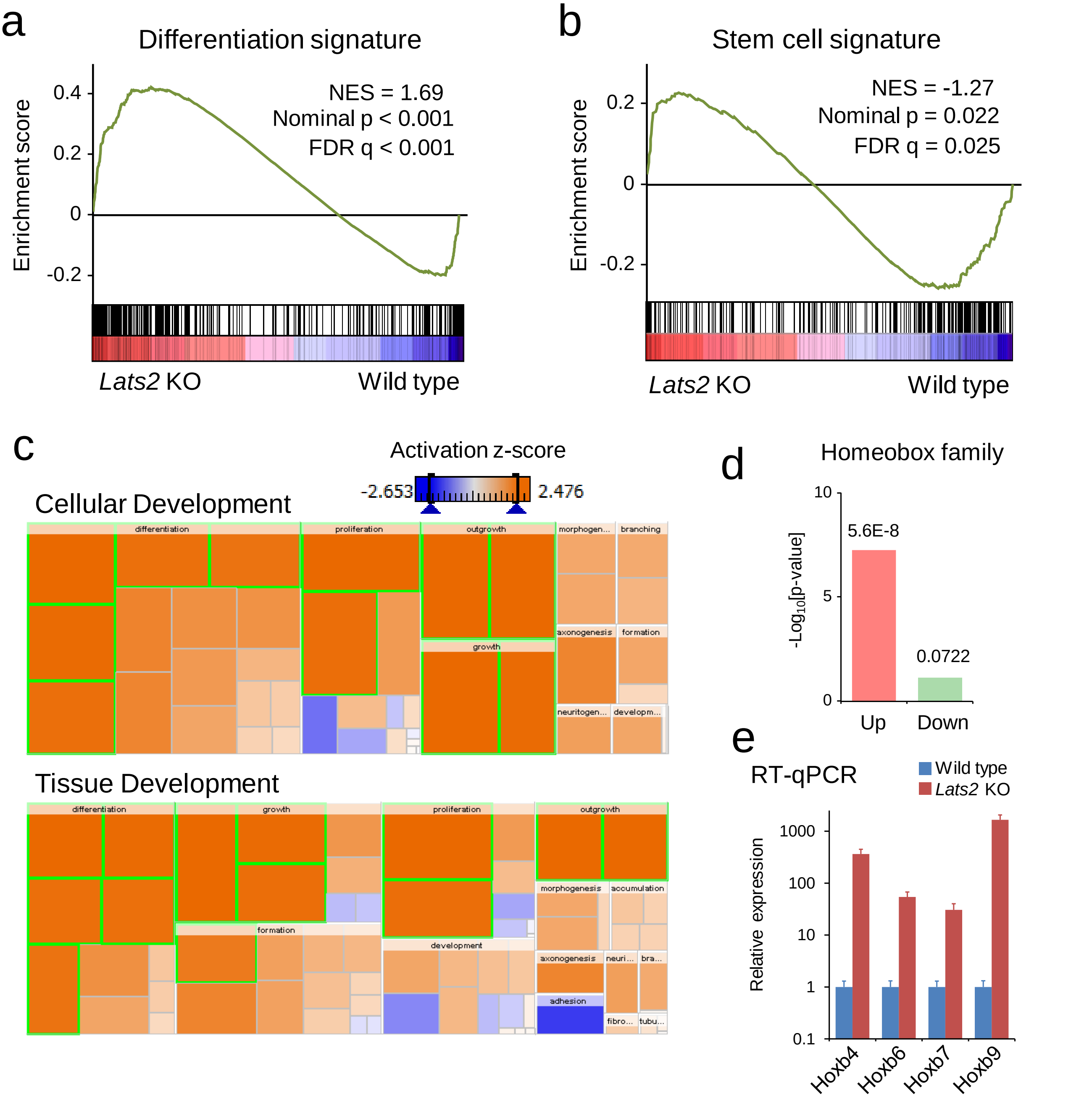

Supplementary Figure I. *Lats2* KO causes de-repression of genes involved in differentiation processes. (**a**–**b**) GSEA of *Lats2* KO MEFs for hES-specific genes (a) and differentiation-related genes (b). Expression changes (all expressing probes in three independent cell lines were combined into a single rank ordered gene list) upon knockout of Lats2 were subjected to GSEA as signature dependent on loss of Lats2. hESC-specific genes were those over-expressed in human ES cells in 5 or more of 20 profiling studies, and differentiation genes were those over-expressed in differentiated cells in 4 or more studies [5]. (**c**) Heatmaps showing facilitation of differentiation processes upon *Lats2* KO. DEGs in *Lats2* KO MEFs (≥2-fold, gene ranked in (a)) were subjected to IPA software. Heat-color represents upregulation of cellular (top) and tissue (bottom) differentiation processes in canonical pathways. (**d**) Enrichment analysis for protein families showing significant overlap of up-regulated genes in Lats2-KO MEFs with Homeobox protein family. DEGs in *Lats2* KO MEFs (≥2-fold, p-value <0.05) were subjected to NextBio analysis. (**e**) Gene expression analysis showing de-repression of *Hoxb* cluster in *Lats2* KO MEFs. RT-qPCR was performed in two independent experiments, and levels of each transcript were normalized to *Gapdh*; error bars show SD.

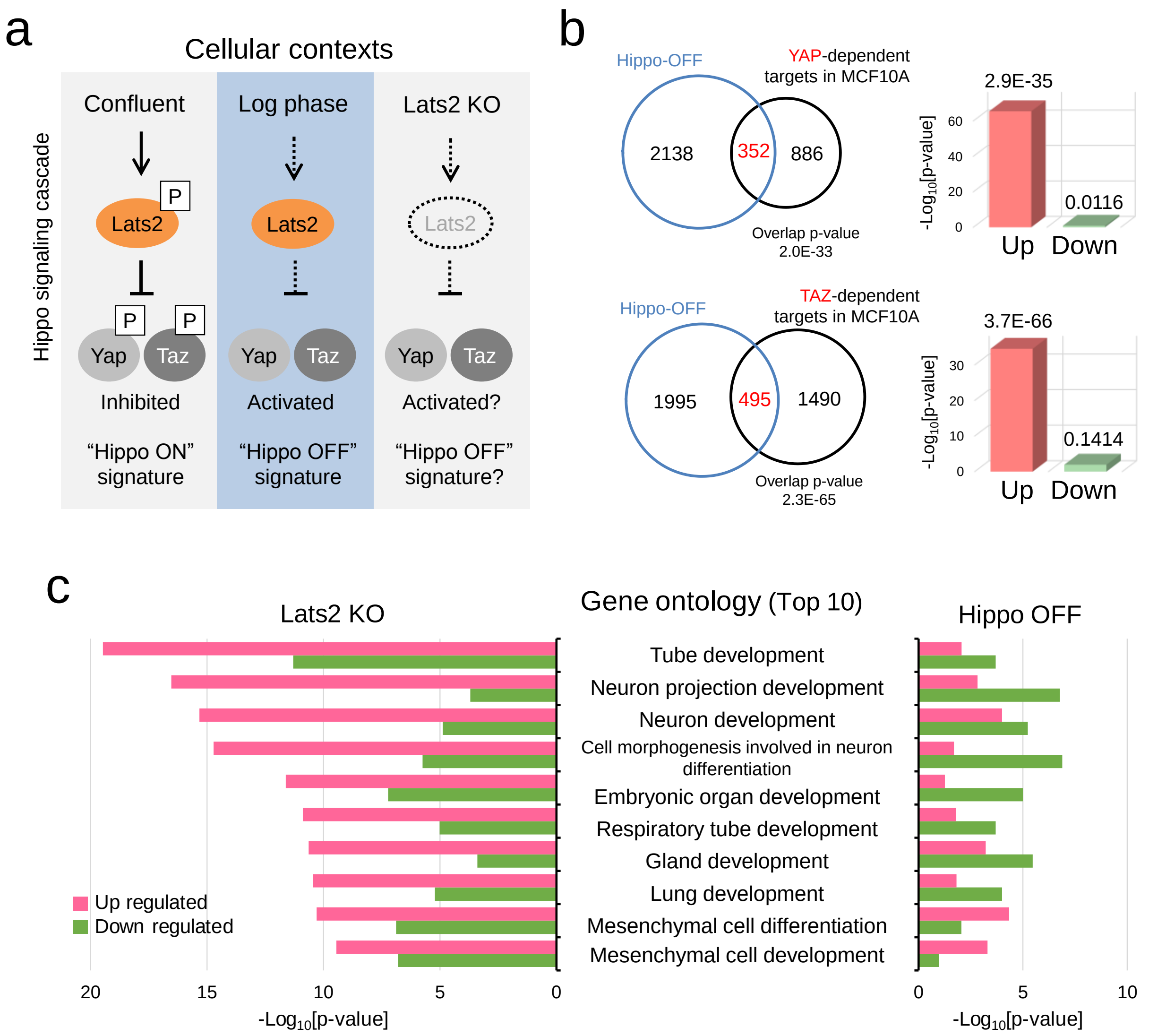

Supplementary Figure J. Correlation of *Lats2* KO profile with canonical Hippo signature. (**a**) Schematic representation of the canonical Hippo signaling cascade and the putative state caused by *Latas2* KO. (**b**) Significant overlap of DEGs in growing MEFs (i.e., Hippo-OFF; Lats2 is not activated) with genes up-regulated upon over-expression of YAP (top) or TAZ (bottom) in MCF10A cells in previous study. DEGs upon contact inhibition (growing vs. confluent) (≥2-fold, p-value <0.05) were subjected to NextBio analysis. Venn diagrams show the number of common and unique genes in both sets. Bar plots show the significance of overlap in each direction. (**c**) Enrichment analysis for canonical pathways related to differentiation processes. DEGs (≥2-fold, p-value <0.05) in *Lats2* KO MEFs and in a Hippo-inactive state (growing in log-phase/high-density culture of wild type MEFs) were subjected to NextBio analysis. *Lats2* KO profile shows associations with differentiation processes more significantly than Yap/Taz-dependent profile. See also supplementary S6 and S7 Table.
